# LiFePO_4_/Nano-LLZTO Composite Cathodes for Enhanced Performance of Solid-State Lithium Batteries

**DOI:** 10.1021/acsami.5c25967

**Published:** 2026-02-27

**Authors:** Jaturon Kumchompoo, Bo-Huei Yang, Jintara Padchasri, Pinit Kidkhunthod, Jyh-Tsung Lee, Chia-Chen Li

**Affiliations:** † Department of Materials Science and Engineering, 34881 National Tsing Hua University, Hsinchu 30013, Taiwan; ‡ Department of Chemistry, 34874 National Sun Yat-Sen University, Kaohsiung 80424, Taiwan; § 530102 Synchrotron Light Research Institute (Public Organization), 111 University Avenue, Muang District, Nakhon Ratchasima, 30000, Thailand; ∥ Department of Medicinal and Applied Chemistry, Kaohsiung Medical University, Kaohsiung 80708, Taiwan

**Keywords:** Solid-state lithium battery, Composite cathode, Conductivity, LLZTO nanoparticles, Numerical simulation

## Abstract

Solid-state lithium batteries (SSLBs) offer improved safety and stability over conventional liquid-electrolyte systems but often suffer from sluggish ion transport and poor interfacial contact within the cathode. To address these limitations, we investigate the incorporation of nanosized Li_6.75_La_3_Zr_1.75_Ta_0.25_O_12_ (nano-LLZTO) particles into a LiFePO_4_ cathode to enhance ionic conductivity and electrochemical performance. Finite element method simulations and experiments reveal that downsizing LLZTO from the microscale to the nanoscale substantially enhances Li^+^ flux uniformity and ionic conductivity (6.48 × 10^–5^ S cm^–1^ vs 1.03 × 10^–5^ S cm^–1^), forming more continuous ion-transport networks. The LiFePO_4_/nano-LLZTO cathode exhibits reduced polarization, higher Coulombic efficiency (99.6%), and superior high-rate capability compared with the microsized LLZTO counterpart, achieving 144 mAh g^–1^ at 1C. Cross-sectional analyses confirm that nano-LLZTO forms homogeneous interfacial coatings, improving ionic percolation and mitigating transport bottlenecks. In situ X-ray absorption near edge structure and extended X-ray absorption fine structure analyses further confirm enhanced redox reversibility and structural stability of Fe sites. Consequently, the LiFePO_4_/nano-LLZTO composite cathode retains 95% of its initial capacity (154 mAh g^–1^) after 200 cycles at 0.2C and 25 °C. This work demonstrates that reducing LLZTO particle size effectively enhances the cathode ion-transport network and ionic conductivity, thereby improving the rate capability and cycling stability of LiFePO_4_-based SSLBs.

## Graphical abstract

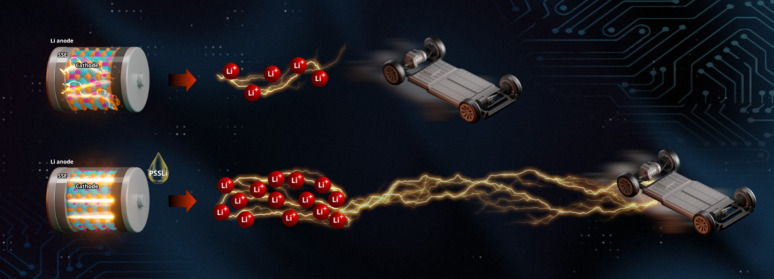

## 1 Introduction

Lithium iron phosphate (LiFePO_4_) is widely recognized as a promising cathode-active material for solid-state lithium-ion batteries (SSLBs), offering several advantages that align well with the demands of next-generation energy storage.^1–3^ This makes it especially well-suited for high-reliability applications such as electric vehicles and stationary energy storage systems, where safety is critical. LiFePO_4_ also exhibits a flat discharge plateau around 3.4 V, providing a consistent voltage output that simplifies battery management and enhances system efficiency.^4–6^ In contrast to layered oxide cathodes, LiFePO_4_ does not release oxygen during cycling, which suppresses interfacial side reactions and improves long-term stability. Its composition, made up of abundant and nontoxic elements such as iron and phosphorus, also makes it environmentally friendly and cost-effective, supporting scalable and sustainable battery production.^7,8^

Despite its favorable characteristics such as thermal stability, safety, and long cycle life, LiFePO_4_ suffers from two intrinsic drawbacks that pose significant challenges for SSLB applications: extremely low electronic conductivity (∼10^–9^ S cm^–1^) and a low lithium-ion diffusion coefficient (*D*_Li_^+^ ∼ 10^–14^ m^2^ s^–1^).^9,10^ In conventional liquid-electrolyte batteries, these limitations can be partially mitigated by the use of conductive additives and the high ionic mobility of the liquid phase. However, in SSLBs—where both ion and electron transport are constrained to solid–solid interfaces—these issues become far more critical. Insufficient ionic and electronic transport results in high interfacial polarization, sluggish redox kinetics, and incomplete utilization of the active material, ultimately compromising both performance and stability.^11^ Therefore, overcoming these transport limitations is crucial to fully realize the potential of electrodes in solid-state systems. One approach involves incorporating solid electrolytes—such as solid polymer electrolytes,^12^ inorganic solid electrolytes,^13^ and polymer/inorganic hybrid electrolytes^14^—into the electrodes to enhance ionic conductivity and improve interfacial contact.

To address this, our previous work demonstrated that incorporating well-dispersed, microsized Li_6.75_La_3_Zr_1.75_Ta_0.25_O_12_ (micro-LLZTO), a fast ion-conducting garnet-type ceramic electrolyte, into the LiFePO_4_ cathode can effectively enhance the ionic conductivity of the composite cathode and improve overall battery performance.^15^ The micro-LLZTO powders were uniformly dispersed on LiFePO_4_ using a dispersant. Building on this insight, this study investigates a more refined strategy using nanosized LLZTO (nano-LLZTO) particles uniformly dispersed within the LiFePO_4_ cathode. Numerical simulations using the finite element method demonstrate that this nanoscale dispersion creates more continuous and percolated Li^+^ transport pathways, leading to further improvement in electrochemical performance compared to microsized additives.^16^ Nano-LLZTO is selected for its advantageous properties including high ionic conductivity, excellent chemical and electrochemical stability, and structural compatibility with LiFePO_4_. Moreover, its relatively high mechanical modulus helps preserve interfacial contact and suppress dendrite growth when paired with lithium metal anodes.^17,18^ By leveraging the nanoscale architecture and material properties of LLZTO, this work aims to mitigate interfacial resistance and ion transport bottlenecks while preserving the intrinsic advantages of LiFePO_4_. A combination of theoretical calculations, electrochemical testing, and in situ spectroscopic analysis demonstrates that the strategic integration of nano-LLZTO into the LiFePO_4_ cathode yields SSLBs with enhanced rate capability, improved stability, and extended cycle life.

## 2 Experimental Section

### 2.1 Preparation of Solid Electrolyte

A ceramic-in-polymer composite solid electrolyte was fabricated using garnet-type LLZTO powder (either nano-LLZTO with an average particle size of 200 nm or micro-LLZTO with a particle size of 6–7 μm; 99.99% purity, MTI Corporation, Richmond, CA) and poly­(vinylidene fluoride-*co*-hexafluoro propylene) (PVDF-HFP, an average molecular weight of ∼400,000 g·mol^–1^, Sigma-Aldrich, USA). The structure and morphology of nano-LLZTO and micro-LLZTO were confirmed by X-ray diffraction (XRD) and scanning electron microscopy (SEM) (see Supporting Information). LLZTO powders were predried at 100 °C for 24 h prior to use to minimize potential dehydrofluorination of PVDF-HFP.^19^ The LLZTO and PVDF-HFP were mixed in a 7:3 mass ratio using a cosolvent of *N*,*N*-dimethylacetamide (DMAC, J.T. Baker, USA) and acetone (1:1 by volume). To improve dispersion and wettability of LLZTO in the polymer matrix, 10 wt % poly­(4-styrenesulfonic acid) lithium salt (PSSLi, 30 wt % aqueous solution, Sigma-Aldrich, USA) based on LLZTO mass was added as a dispersant. The mixture was homogenized for 30 min at 400 rpm using a 3D ball mixer (400S, Chia Mey Machinery Co. Ltd., Taiwan), then cast onto a Teflon plate and dried in a vacuum oven at 80 °C for 12 h to give either micro-LLZTO/PVDF-HFP or nano-LLZTO/PVDF-HFP composite electrolyte.

### 2.2 Preparation of Composite Cathodes

In the preparation of the LiFePO_4_/nano-LLZTO composite cathode, LiFePO_4_ powder (M12, Advanced Lithium Electrochemistry Co., Ltd., Taiwan) with a median size of 2.4 μm was mixed with poly­(vinylidene fluoride) (PVDF, Alfa Aesar, USA) as a binder, carbon black (Super-P, Timcal A+G Sins, Switzerland) and graphite (KS6, Timcal A+G Sins, Switzerland) as electron-conductive agents, LLZTO powder (either nano-LLZTO (∼200 nm) or micro-LLZTO (6–7 μm)) as a lithium-ion-conductive additive, and PSSLi as a dispersant,^15^ using *N*-methyl-2-pyrrolidone (NMP, J.T. Baker, USA) as the solvent. The mixture was homogenized using a 3D ball mixer at 1,000 rpm for 1 h. The resulting cathode slurry was cast onto aluminum foil and dried in a vacuum oven (HONDWEN, DOV30, Taiwan) at 80 °C under 10^–3^ Torr for 12 h. The weight ratio of LiFePO_4_, Super-P, KS6, PVDF, LLZTO, and PSSLi in the dried cathode was 81.1:4.1:1.4:4.6:8.1:0.8. In the case of the pristine LiFePO_4_ electrode, LLZTO and PSSLi were not included. All dried cathodes were calendered to an average thickness of 80 ± 5 μm, with an active material areal loading of approximately 5 mg cm^–2^.

### 2.3 Assembly of Coin Cells

Electrochemical testing was conducted with CR2032 coin cells, assembled in an argon-filled glovebox where water and oxygen levels were kept below 0.5 ppm. Lithium foil was used as the anode, while either the LiFePO_4_/nano-LLZTO composite or the pristine LiFePO_4_ electrode served as the cathode. The nano-LLZTO/PVDF-HFP composite electrolyte was employed as the separator. For cells with the LiFePO_4_/micro-LLZTO cathode, the micro-LLZTO/PVDF-HFP composite electrolyte was used as the separator. To enhance interfacial contact, a small quantity (4 μL) of liquid electrolyte was applied at the interface between the electrodes and the solid electrolyte. This electrolyte consisted of 1.0 M LiPF_6_ (Tomiyama Pure Chemical Industries, Japan) in a cosolvent mixture of ethylene carbonate, propylene carbonate, and diethyl carbonate at a volume ratio of 3:2:5 (all solvents from Alfa Aesar, USA).

### 2.4 Characterizations

XRD was performed using Bruker D2 Phaser to identify the crystal structure and phase composition of the LLZTOs. The microstructures and elemental compositions of the LLZTO, composite cathodes and composite solid electrolyte were characterized using a SEM (SU8010, Hitachi, Japan) equipped with an energy-dispersive X-ray spectroscopy (EDS) system. Galvanostatic charge–discharge tests were performed at 25 °C using a battery testing system (CT2001A, LANHE, China), with a constant cycling rate of 0.5C and variable rates ranging from 0.1C to 1.0C, within a voltage window of 2.5–4.2 V. The *D*_Li_^+^ was determined via the galvanostatic intermittent titration technique (GITT), which involved applying current pulses at 0.2C for 10 min, each followed by a 1 h rest period. Cyclic voltammetry (CV) measurements were carried out using an electrochemical workstation (Multi PalmSens4, PalmSens, Netherlands) over the same voltage range (2.5–4.2 V) at a scan rate of 0.1 mV s^–1^. Electrochemical impedance spectroscopy (EIS) was also performed using the same system, with a frequency range of 10^5^ to 10^–1^ Hz and a perturbation amplitude of 10 mV. To analyze the EIS data, the distribution of relaxation times (DRT) was calculated using MATLAB-based open-source software. Equivalent circuit models were determined by fitting both real and imaginary components of the impedance spectra (excluding inductive elements) using Tikhonov regularization and a nonlinear least-squares approach. The DRT was discretized using the Gaussian method, with a full width at half-maximum of 0.5, and second-order derivative regularization with a regularization parameter of 0.001. Since the composite electrolyte is based on a flexible PVDF-HFP polymer matrix, no external stack pressure was applied during all of electrochemical measurements. The coin cell assembly itself ensures sufficient interfacial contact. For the numerical analysis, Li^+^ transport in the composite cathode was simulated using the finite element method (FEM) in COMSOL Multiphysics (version 5.6), with the simulation parameters summarized in Table S1. Two-dimensional battery models were developed and solved using coupled interfaces from the battery design module and chemical reaction engineering module. In situ X-ray absorption near edge structure (XANES) and extended X-ray absorption fine structure (EXAFS) measurements were conducted using an X-ray spectrometer at beamline BL5.2 (X-ray absorption spectroscopy) at the Synchrotron Light Research Institute, Thailand. Cross-sectional imaging of the LiFePO_4_/LLZTO electrodes was carried out using a laser-assisted cryogenic focused ion beam–scanning electron microscopy (Laser-Cryo-FIB-SEM) system (Crossbeam 550, Zeiss). Prior to milling, the electrodes were coated with a thin platinum layer by sputtering at a current of 10 mA for 60 s to prevent surface charging. To preserve the pristine structural integrity and chemical state, the samples were maintained at a cryogenic temperature of –180 °C during ion milling. The Ga^+^ ion beam was operated at 30 kV with a beam current of 1.5 nA for coarse milling to a depth of ∼15 μm, followed by fine polishing at 300 pA to obtain a smooth cross-sectional surface. Subsequent *in situ* SEM imaging was performed at 5 kV, and EDS was conducted at 10 kV for elemental mapping.

## 3 Results and Discussion

### 3.1 Effect of LLZTO Particle Size on Li^+^ Transport

It is recognized that incorporating ion-conductive materials into the cathode can theoretically enhance Li^+^ transport and, consequently, improve the performance of SSLBs.^20^ In this study, LLZTO was chosen as an additive due to its excellent electrochemical stability, favorable ionic conductivity, and good interfacial compatibility with a ceramic-in-polymer solid electrolyte made of LLZTO and PVDF-HFP.^21,22^ To determine whether nano- or micro-LLZTO particles are more effective for improving the cathode performance, FEM simulations were carried out to preliminarily assess the effect of LLZTO particle size on Li^+^ transport within the composite cathode, as shown in Figure 1 (Figure S1 and Movies S1–S3). The particle sizes of the micro- and nano-LLZTO were set to 5 μm and 200 nm, respectively. Figure 1a–c illustrates the simulated Li^+^ flux distributions in cathodes composed of pristine LiFePO_4_, LiFePO_4_ with micro-LLZTO, and LiFePO_4_ with nano-LLZTO, respectively, after discharging at 0.2C for 400 s. It is noteworthy that, for simplification, the simulations assumed that Li^+^ transport was unaffected by electrochemical reactions and that the cathode was composed solely of LiFePO_4_ and LLZTO, excluding other additives such as conductive agents and interfacial substances like lithium carbonates.^23,24^

**Figure 1:**
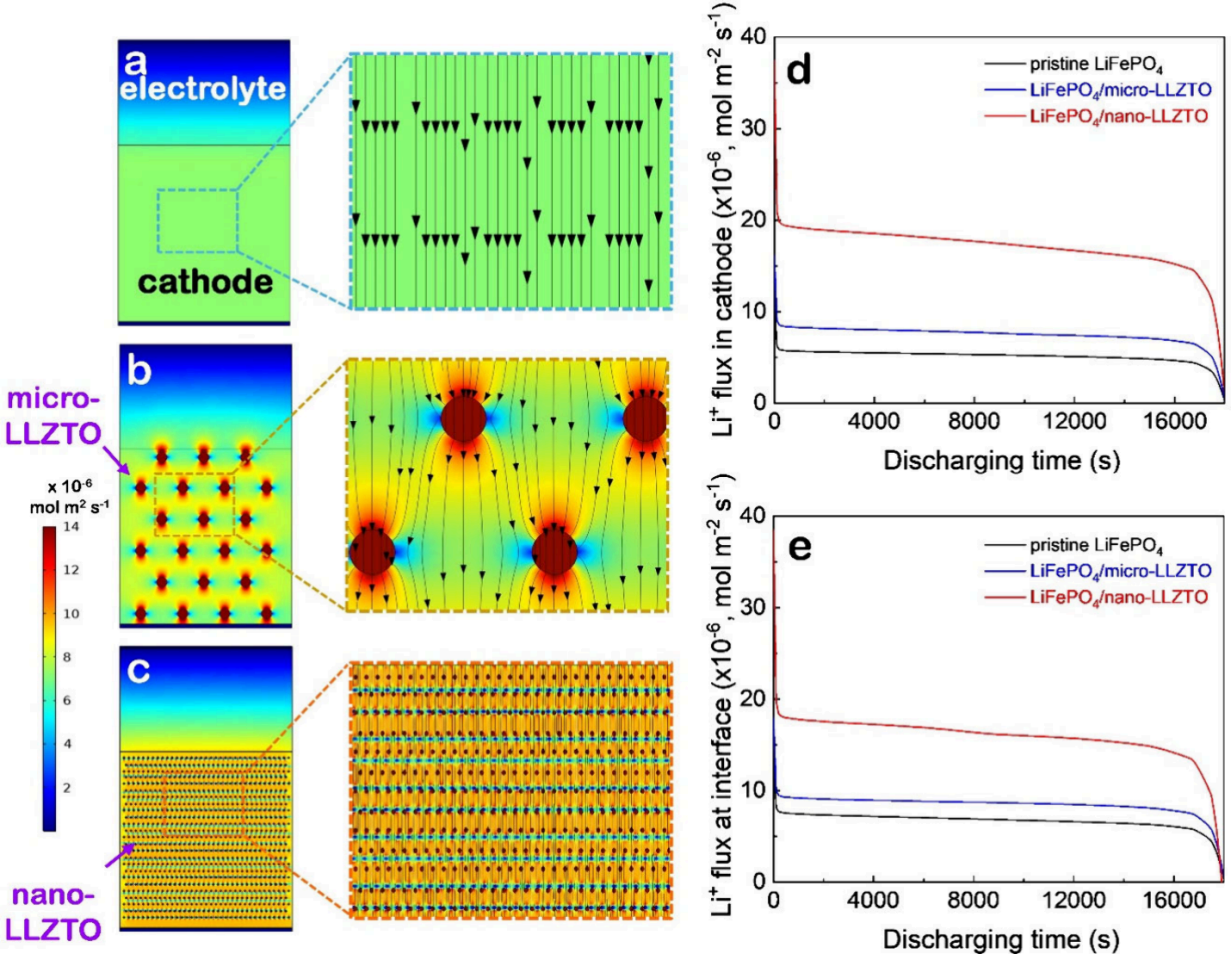
Finite element method-simulated Li^+^ flux distributions in solid-state batteries with (a) a pristine LiFePO_4_ cathode, (b) a composite LiFePO_4_ cathode with micro-LLZTO particles, and (c) a composite LiFePO_4_ cathode with nano-LLZTO particles after 400 s of discharge. Two composite cathodes have the same volume of added LLZTO particles. Magnified views are shown on the right. Simulated Li^+^ flux over time in (d) the entire cathode region and (e) the cathode–electrolyte interface. Discharge rate: 0.2C.

The color maps and flux streamlines in Figure 1a–c, along with their magnified views, clearly demonstrate that incorporating either micro- or nano-LLZTO particles enhances Li^+^ transport, with nano-LLZTO incorporation exhibiting the most uniform and intense Li^+^ flux throughout the cathode. The superior performance of the nano-LLZTO system, compared to the micro-LLZTO counterpart, is primarily attributed to the formation of more continuous and efficient percolation pathways by the nano-LLZTO particles, which shorten diffusion distances and facilitate more effective Li^+^ transport. Quantitative results in Figure 1d and e further confirm these findings: both the average Li^+^ flux within the cathode and at the cathode–electrolyte interface are significantly higher in the LiFePO_4_/micro-LLZTO system. Notably, the average flux in the LiFePO_4_/nano-LLZTO cathode (Figure 1d) is nearly double that of the LiFePO_4_/micro-LLZTO cathode, attributed not only to the enhanced interfacial flux at the cathode–electrolyte interface (Figure 1e) but also to the increased Li^+^ flux through both the LiFePO_4_ matrix and the nano-LLZTO particles (Figure S2). These results collectively demonstrate the clear advantage of nano-LLZTO fillers in facilitating efficient ionic transport and improving interfacial contact in SSLBs.

### 3.2 Battery Cyclability

Since the results in Figure 1 indicate that incorporating nano-LLZTO particles significantly enhances the ionic conductivity of the composite cathode, a battery employing a LiFePO_4_ cathode with nano-LLZTO particles (200 nm) (LiFePO_4_/nano-LLZTO) was assembled and tested under charge–discharge conditions at a constant rate of 0.2C between 2.5 and 4.2 V at 25 °C. Notably, the calculation results of Figure 1 rely on uniformly distributed LLZTO particles, so the composite cathodes fabricated in this investigation are made with well-dispersed nano-LLZTO particles, achieved through a small addition of the effective dispersant, PSSLi (Figure S6).^15^ The cycling performance was compared with that of a control cell using a pristine LiFePO_4_ cathode without LLZTO, as shown in Figure 2a. Notably, the nano-LLZTO powder was thermally pretreated at 1000 °C in an Ar atmosphere to remove surface Li_2_CO_3_, which commonly forms during storage.^25^

**Figure 2:**
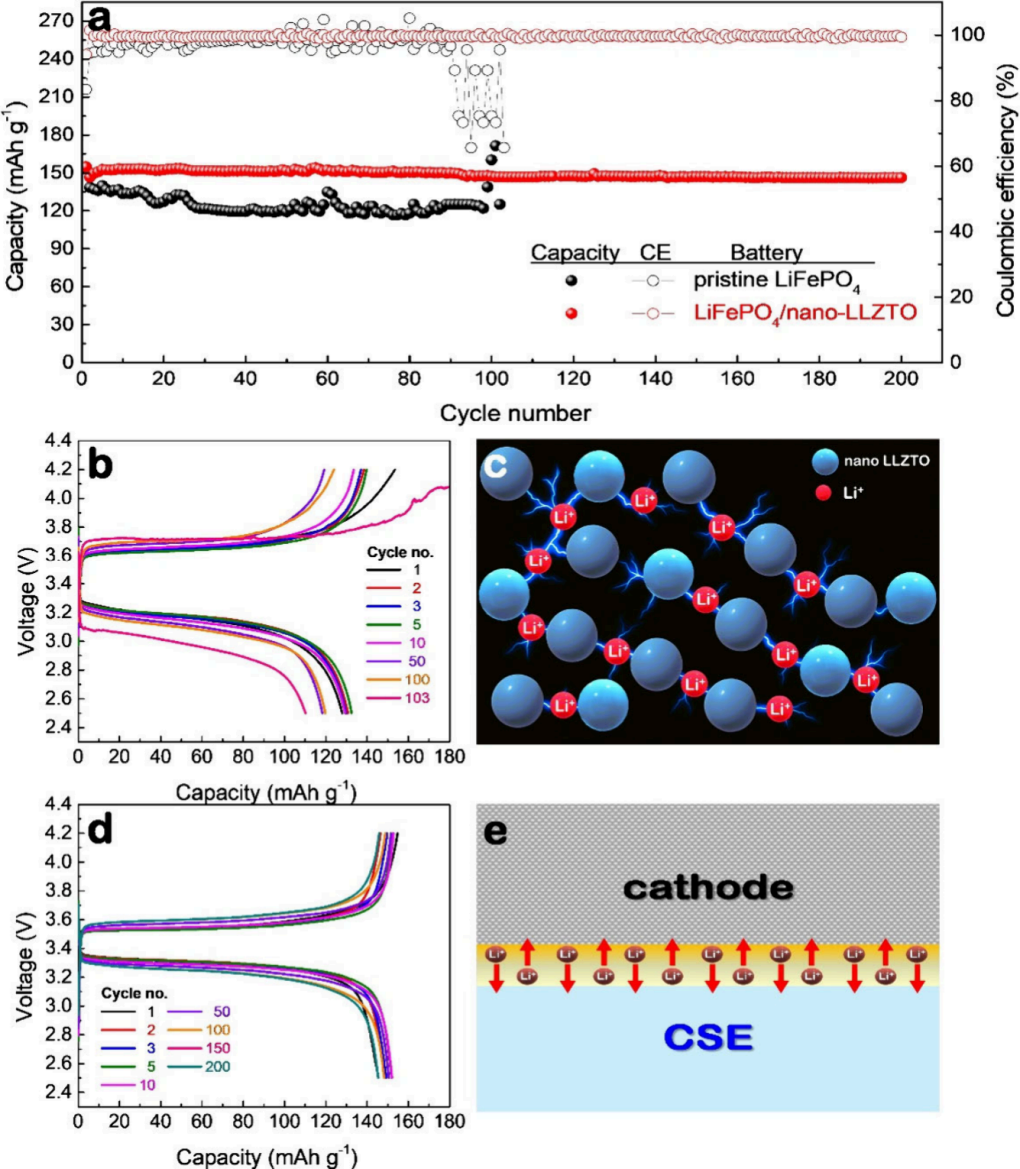
(a) Cycling performance of SSLBs with pristine LiFePO_4_ and LiFePO_4_/nano-LLZTO composite cathodes at 0.2C. (b, d) Corresponding galvanostatic charge–discharge profiles for SSLBs using (b) pristine LiFePO_4_ and (e) LiFePO_4_/nano-LLZTO cathodes. (c, e) Schematic illustrations of the proposed mechanisms for enhanced conductivity (c) within the composite cathode and (e) at the interface between the cathode and the composite solid electrolyte (CSE).

For the pristine LiFePO_4_ cell, the initial discharge capacity was 153 mAh g^–1^, which decreased to 138 mAh g^–1^ after the first cycle. This capacity is lower than what is typically achieved in liquid electrolyte systems. Moreover, the cycling performance was limited, with the cell failing by the 103rd cycle. Both the reduced capacity and poor cycle life can be attributed to the low ionic conductivity of the LLZTO/PVDF-HFP composite solid electrolyte (6.48 × 10^–5^ S m^–1^), measured by EIS (Figure S10 and Table S3), which is markedly lower than that of conventional liquid electrolyte systems (∼10^–2^ S m^–1^).^26^ This observation aligns with the general trend in SSLBs, which often suffer from limited cycling stability due to the rigid and complex nature of solid–solid interfaces that lack the conformal contact and self-healing behavior of liquid electrolyte systems.^27,28^ Mechanical degradation from repeated lithiation and delithiation at the cathode–composite solid electrolyte interface can lead to interfacial contact loss and increased resistance. Additionally, the absence of a liquid phase hinders Li^+^ transport between the solid electrolyte and the poorly ion-conductive LiFePO_4_ matrix, making it difficult to maintain continuous and uniform ionic pathways. These coupled effects accelerate interfacial degradation, promote space charge layer formation,^29,30^ and ultimately impede Li^+^ mobility, resulting in rapid capacity fade and early cell failure. Notably, these trends are consistent with the charge–discharge profiles recorded during cycling (Figure 2b), which reveal a significant increase in polarization, further confirming the presence of transport limitations and growing interfacial resistance over time.

In contrast, when the composite LiFePO_4_/nano-LLZTO cathode was used, the SSLB delivered an initial capacity of 155 mAh g^–1^, which decreased only slightly to 147 mAh g^–1^ after 200 charge–discharge cycles, demonstrating stable cyclability with 95% capacity retention. This marked improvement is attributed to the enhanced Li^+^ transport facilitated by the nano-LLZTO particles, which form continuous and efficient ionic percolation pathways throughout the cathode, as discussed earlier and illustrated in Figure 2c. Moreover, the simulation results in Figure 1d and e indicate that nano-LLZTO should have offered superior interfacial contact with both the LiFePO_4_ particles and the solid electrolyte, thereby reducing interfacial resistance and enabling more efficient Li^+^ transport within the cathode and across the cathode–composite solid electrolyte interface, as illustrated in Figure 2e. In addition, the resulting uniform ion distribution and stable electrochemical environment likely suppress the formation of space-charge layers and interfacial delamination, allowing the battery to retain both structural and electrochemical integrity over prolonged cycling, as supported by postcycling microstructural analysis of the cathode surface (Figures S11–S14).^31^ Although minor surface roughening is observed after long-term cycling, no severe cracking or phase segregation occurs, indicating that the nano-LLZTO-decorated cathode maintains sufficient mechanical integrity and solid–solid interfacial contact. These favorable effects are also reflected in the charge–discharge profiles during cycling (Figure 2d), which show minimal polarization and nearly overlapping voltage curves, indicating sustained low resistance and stable cathode–composite solid electrolyte interfaces throughout long-term operation.

### 3.3 Electrochemical Redox Behavior

To examine whether the electrochemical behavior of the LiFePO_4_ cathode is affected by the incorporation of LLZTO nanoparticles, Figure 3a and b compares the CV curves of batteries assembled with a pristine LiFePO_4_ cathode and an LiFePO_4_/nano-LLZTO composite cathode over the first five scans within the potential window of 2.5–4.2 V at a scan rate of 0.1 mV s^–1^ vs Li/Li^+^. For both batteries, a single pair of redox peaks is observed, indicating that no side reactions occur upon the inclusion of nano-LLZTO. Notably, the CV curve of the pristine LiFePO_4_ battery exhibits a large voltage gap (ΔV) of 0.511 V between the anodic and cathodic peaks (Figure 3a), indicating significant concentration polarization. This behavior is consistent with the significant polarization observed in its charge–discharge profiles (Figure 2b). In contrast, the LiFePO_4_/nano-LLZTO battery displays a considerably smaller ΔV of 0.488 V (Figure 3b), suggesting reduced polarization as a result of enhanced ionic transport. This observation corroborates the earlier results shown in Figure 2d, where the LiFePO_4_/nano-LLZTO battery exhibited less Li^+^ polarization during cycling.

**Figure 3:**
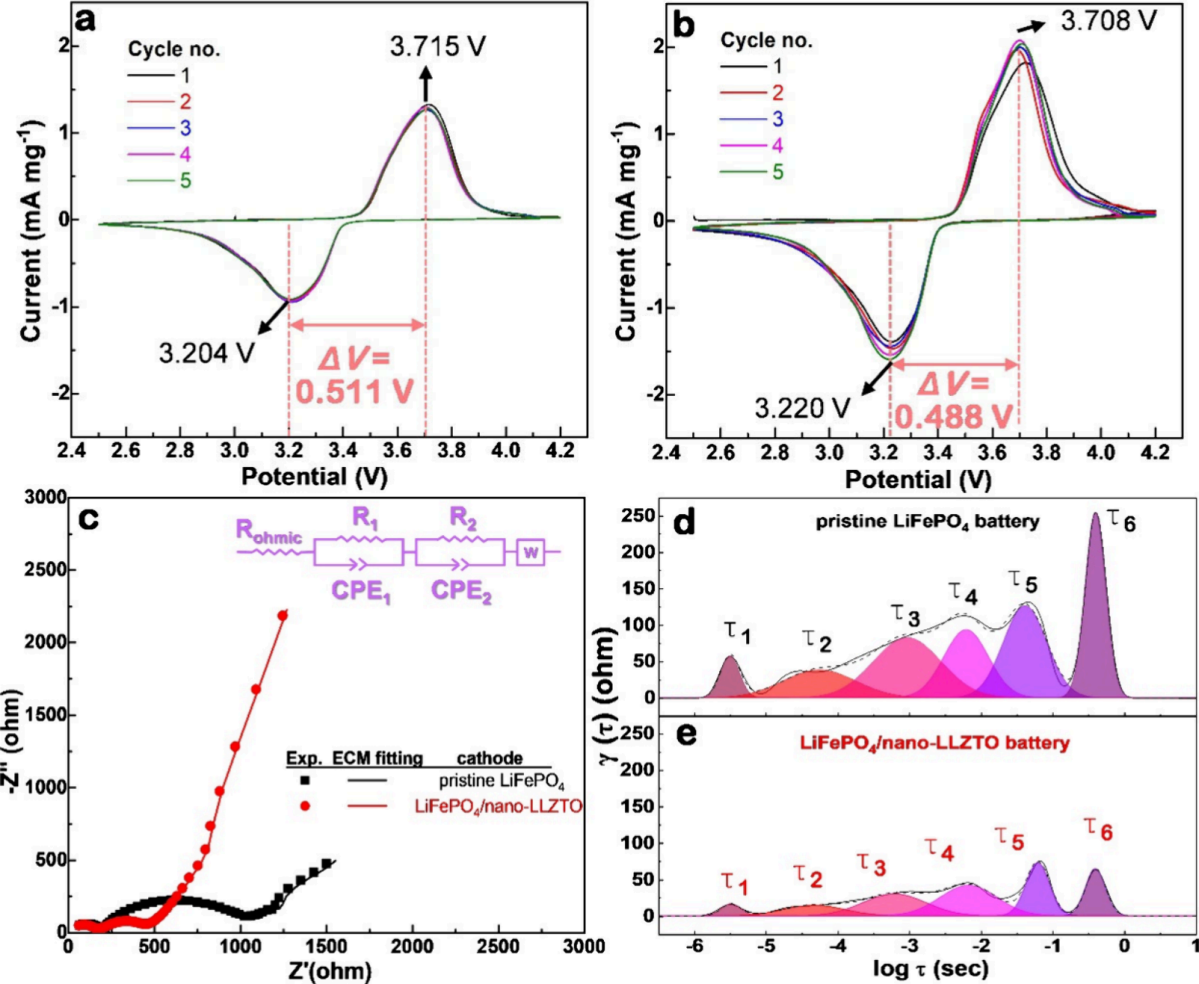
CV curves of SSLBs with (a) pristine LiFePO_4_ and (b) LiFePO_4_/nano-LLZTO composite cathodes. (c) EIS spectra of the corresponding cells shown in (a) and (b). (d, e) DRT analyses of the impedance data for SSLBs with (d) pristine LiFePO_4_ and (e) LiFePO_4_/nano-LLZTO cathodes.

In terms of the peak currents in the CV curves (Figure 3a and b), the solid-state battery with the pristine LiFePO_4_ cathode exhibits significantly lower values compared to the battery using the LiFePO_4_/nano-LLZTO composite cathode. The peak currents at 3.7 V represent the number of charges (or electrons) involved in the electrochemical reaction and is primarily influenced by the concentration (*C*, mol cm^–3^) and diffusion coefficient (*D*, cm^2^ s^–1^) of the electroactive species, as described by the Randles–Sevcik equation:^32^

1 $$i_{p}=0.4463nFAC{\left(\frac{nF\upsilon D}{RT}\right)}^{1/2}$$

where *A* is the electrode area (cm^2^), *F* is the Faraday constant (C mol^–1^), υ is the scan rate (V s^–1^), *R* is the gas constant (J K^–1^ mol^–1^), and *T* is the temperature (K). A higher peak current typically reflects improved ionic and electronic conductivity. Evidently, the elevated peak current observed in the battery with the composite cathode indicates that the incorporation of nano-LLZTO substantially enhances electrode conductivity. This enhancement is one of the key contributors to the significantly improved electrochemical performance of the solid-state batteries shown in Figure 2a.

To further assess the conductivity of the cathodes, AC impedance measurements were conducted on the solid-state batteries before cycling. Figure 3c shows the Nyquist plots of EIS for the cells assembled with pristine LiFePO_4_ and composite LiFePO_4_/nano-LLZTO cathodes. Both spectra display a depressed semicircle in the high- to midfrequency region, corresponding to interfacial charge-transfer resistance at the electrode–electrolyte interface. Notably, the semicircle diameter for the pristine LiFePO_4_ cell (black) is substantially larger than that of the composite cathode cell (red), indicating greater interfacial resistance and slower charge-transfer kinetics. This observation aligns with the earlier results in Figure 2a, where the pristine LiFePO_4_ cell exhibited lower electrochemical performance and shorter cycle life, likely due to insufficient ionic transport pathways and poor interfacial contact with the solid electrolyte. In the low-frequency region, the red curve associated with the composite cathode exhibits a steeper inclined tail compared to the black curve, indicating stronger Warburg-type behavior. This tail reflects the diffusion of Li^+^ within the electrode and electrolyte. The more extended tail in the red curve suggests improved ionic conductivity and enhanced ion diffusion dynamics, attributable to the presence of nano-LLZTO particles. These particles promote continuous ionic percolation networks and better interfacial contact between the cathode and the composite solid electrolyte. In contrast, the pristine LiFePO_4_ cathode demonstrates a shorter, more compressed tail, implying hindered Li^+^ diffusion and less capacitive response. Overall, the EIS results confirm that incorporating nano-LLZTO in the cathode significantly improves interfacial and transport properties, which correlates well with the enhanced cycling stability observed earlier.

Since the Nyquist plots in Figure 3c exhibit overlapping or poorly defined semicircles, the DRT method was employed to mathematically deconvolute the EIS data from the frequency domain into the time constant domain.^33–35^ This transformation enables a more resolved interpretation of individual electrochemical processes, such as bulk resistance, grain boundary resistance, charge-transfer resistance, solid–solid interfacial impedance, and Li^+^ diffusion impedance, all of which collectively contribute to the total cell impedance. Based on the DRT spectra shown in Figure 3d and e (Figure S15), a comprehensive comparison of impedance relaxation processes for the solid-state batteries with pristine LiFePO_4_ and composite LiFePO_4_/nano-LLZTO cathodes can be made. In DRT analysis, each peak corresponds to a distinct electrochemical or physical process with a characteristic relaxation time constant (τ). The number, position, and intensity of these peaks offer valuable insight into the resistive and capacitive behavior of the battery components. For the pristine LiFePO_4_ battery (Figure 3d), six broad and partially overlapping peaks are observed across a wide range of relaxation times. This indicates the presence of multiple resistive processes, particularly substantial contributions from charge-transfer and diffusion-related resistances. The large integrated area under the peaks, especially those spanning intermediate to long relaxation times (e.g., τ_3_ to τ_6_), suggests significant interfacial and ion transport limitations. These features are consistent with the high interfacial impedance observed in the Nyquist plot and the notable voltage polarization (ΔV) observed in the CV profiles. The broad, unresolved peaks also point to sluggish Li^+^ transport dynamics and the lack of well-defined ion pathways in the pristine LiFePO_4_ cathode. In contrast, the DRT profile of the LiFePO_4_/nano-LLZTO battery (Figure 3e) also exhibits six peaks, but with markedly lower intensities and reduced total area, especially in the mid- and high-τ regions. Peaks τ_1_ and τ_2_ emerge at shorter relaxation times with low intensity, indicating fast and efficient Li^+^ transport through the bulk and grain boundary, likely facilitated by the incorporation of nano-LLZTO. The significantly attenuated τ_3_ to τ_5_ peaks reflect a reduction in interfacial resistance and improved contact between the cathode and the composite solid electrolyte. These DRT findings are in excellent agreement with the lower overall impedance and narrower ΔV previously observed, confirming that the inclusion of nano-LLZTO effectively enhances ionic percolation, mitigates interfacial impedance, and improves charge-transfer kinetics in the solid-state battery.

### 3.4 Improved LiFePO_4_ Conversion during Redox in Composite Cathode

Given the improved ionic conductivity of the composite LiFePO_4_/nano-LLZTO cathode, it is reasonable to expect enhanced electrochemical reaction kinetics and more complete utilization of the active LiFePO_4_ material, ultimately benefiting the overall performance of the SSLB. To further examine this possibility, in situ XANES and EXAFS analyses were performed to probe the local electronic structure and coordination environment of the Fe center during battery operation, as shown in Figures 4 and 5, respectively. These techniques enable real-time monitoring of redox behavior and structural evolution, providing direct insight into the electrochemical activity and reversibility in both the pristine and composite cathode systems. To investigate the local structural evolution of Fe during electrochemical cycling, in situ Fe K-edge EXAFS analysis was performed on pristine LiFePO_4_ and LiFePO_4_/nano-LLZTO composite cathodes. The custom-designed coin cell included a 2 mm hole aligned with the cathode region, sealed with Kapton tape and epoxy resin, allowing X-rays to pass exclusively through the LiFePO_4_ material without interference from Li metal, as shown in Figure 4a. Figure 4b-e presents the in situ Fe K-edge XANES spectra collected during the charge and discharge processes of SSLBs with pristine LiFePO_4_ (Figure 4b and c) and LiFePO_4_/nano-LLZTO composite (Figure 4d and e) cathodes. In both systems, the Fe absorption edge shifts between the Fe^2+^ and Fe^3+^ reference positions (FeO and Fe_2_O_3_), corresponding to the Fe^2+^/Fe^3+^ redox reaction of the LiFePO_4_ active material. This reaction involves the reversible extraction and insertion of Li^+^ ions and electrons, following:^36^

2 $${LiFePO}_{4}⇌{FePO}_{4}+{Li}^{+}+e^{-}$$

**Figure 4:**
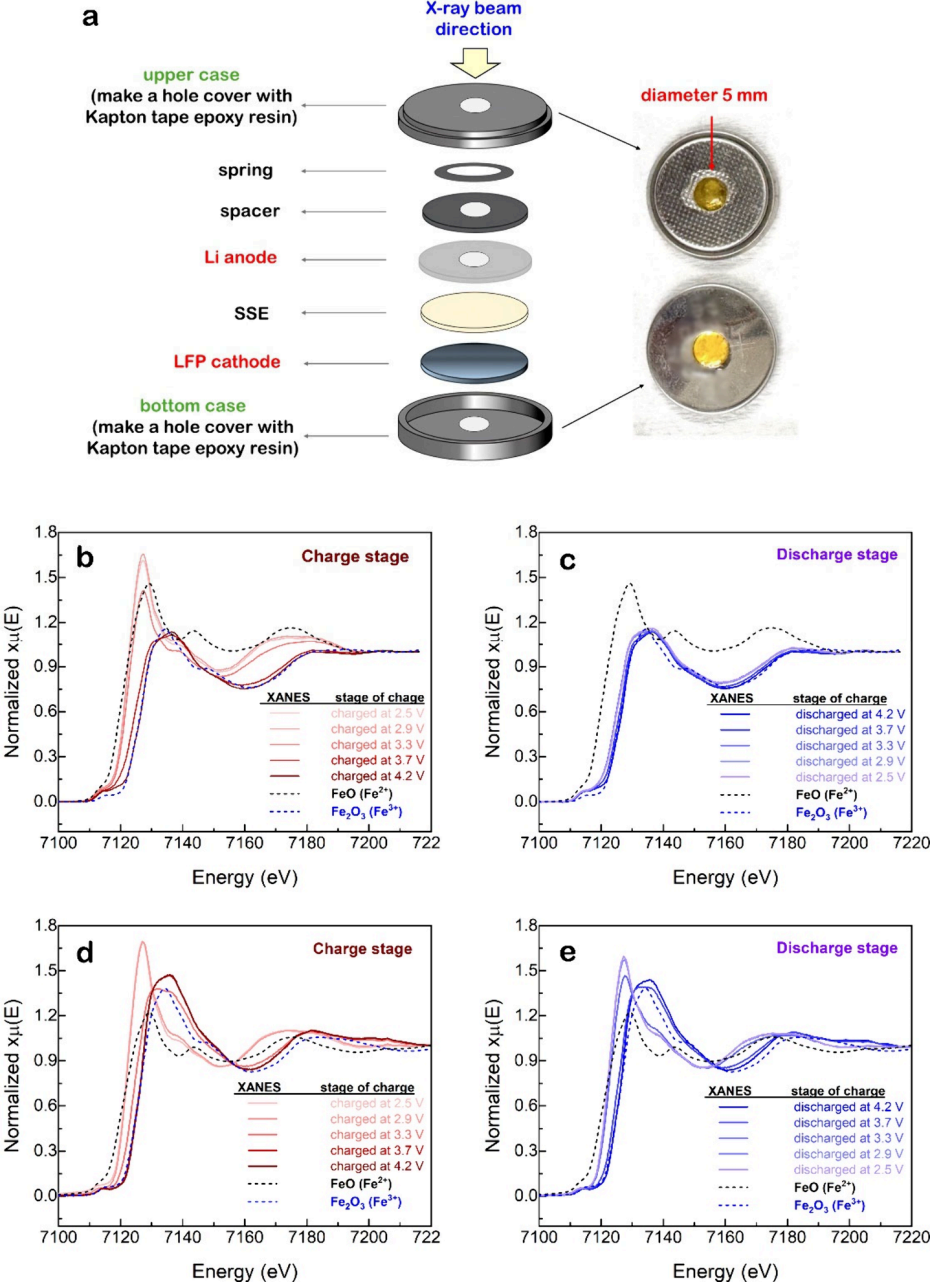
(a) CR2032 cell configuration for in situ XANES measurements. In situ XANES spectra of (b, c) pristine LiFePO_4_ and (d, e) LiFePO_4_/nano-LLZTO cathodes during (b, d) charging and (c, e) discharging at a rate of 0.2C.

**Figure 5:**
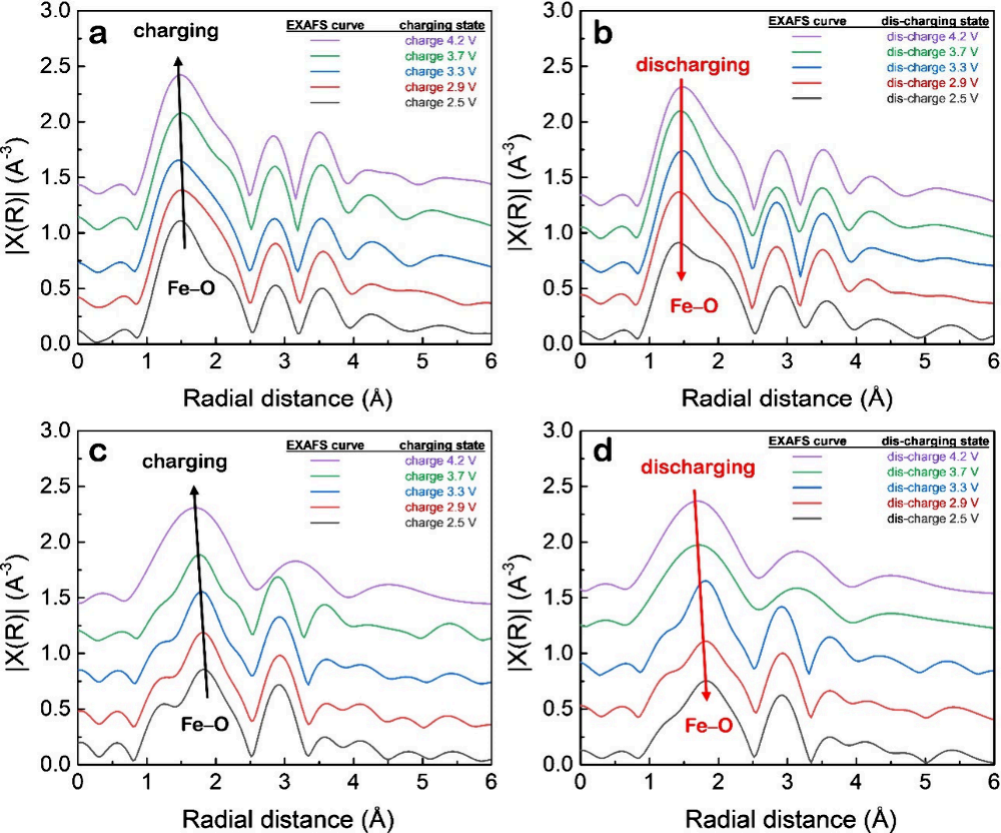
In situ EXAFS spectra of (a, b) pristine LiFePO_4_ and (c, d) LiFePO_4_/nano-LLZTO cathodes during (a, c) charging and (b, d) discharging at different voltages.

During delithiation (charging), Li^+^ ions are extracted from the LiFePO_4_ structure, and Fe^2+^ is oxidized to Fe^3+^, forming FePO_4_.^37,38^ This is reflected by the shift of the Fe K-edge to higher energy in Figure 4b and d. Ideally, this transformation proceeds as a two-phase reaction in which LiFePO_4_ and FePO_4_ coexist with a moving phase boundary. The Fe K-edge XANES spectra reveal a pronounced difference in white-line intensity between LiFePO_4_ and FePO_4_ (Figures 4b and 4d). The stronger white-line feature observed for LiFePO_4_ originates from its intrinsic electronic structure. In LiFePO_4_, Fe exists predominantly as high-spin Fe^2+^ (3d^6^), which provides a higher density of unoccupied Fe 4p states available for the 1s → 4p transition, resulting in enhanced white-line intensity. In contrast, FePO_4_ contains high-spin Fe^3+^ (3d^5^), in which stronger Fe–O covalent interactions and increased Fe 3d–O 2p hybridization reduce the transition probability to Fe 4p states, leading to a weaker white-line feature. This interpretation is consistent with previous XANES studies reported in the literature.^39^

During the discharge process, the Fe K-edge XANES spectra of the pristine LiFePO_4_ cathode (Figure 4c) do not fully recover to the Fe^2+^ reference state even at a discharge potential of 2.5 V. Although the spectral contribution from Fe^3+^ species is substantially diminished upon discharge, the edge position remains at an intermediate energy, indicating incomplete electrochemical reduction and the presence of a mixed Fe^2+^/Fe^3+^ state within the X-ray probed region. This behavior can be attributed to the configuration of the in situ XANES cell (Figure 4a). To enhance X-ray transmission, a small hole was introduced in the Li-metal anode, which weakens the electrochemical correspondence between the illuminated cathode region and the Li anode and may result in spatially nonuniform lithium transport during discharge. Consequently, part of the FePO_4_ phase in the probed region is not fully reduced, leading to the absence of a complete edge shift to lower energy. In contrast, the LiFePO_4_/nano-LLZTO composite cathode (Figure 4e) exhibits a distinct shift of the Fe K-edge to lower energy upon discharge, indicating more complete reduction to Fe^2+^. This result suggests that incorporation of nano-LLZTO enhances ionic conductivity and facilitates more homogeneous Li^+^ transport throughout the composite cathode, thereby improving the redox kinetics and utilization of the active material.

Figure 5 presents the Fourier-transformed (FT) EXAFS spectra in R-space. Figure 5a and b show the spectra of pristine LiFePO_4_ cathode during charging and discharging, respectively; Figure 5c and d correspond to the LiFePO_4_/nano-LLZTO composite cathode during charging and discharging, respectively. During charging (Figure 5a), the first major peak—primarily corresponding to the Fe–O coordination shell—shows a gradual decrease in intensity and a slight shift toward lower radial distance.^40^ This trend is consistent with the oxidation of Fe^2+^ to Fe^3+^, resulting in a shorter Fe–O bond and a more compact local structure.^41^ However, during the subsequent discharge (Figure 5b), the Fe–O peak only partially recovers its original intensity and position. This incomplete reversibility suggests that part of the Fe remains in the oxidized Fe^3+^ state or that local structural distortions (such as asymmetric FeO_6_ coordination) persist even after Li^+^ reinsertion.^42^ These irreversible changes in the local environment may contribute to capacity fading and performance degradation over cycling. A markedly different behavior is observed for the LiFePO_4_ electrode containing nano-LLZTO. During charging (Figure 5c), similar trends in Fe–O peak reduction are seen, indicating comparable Fe oxidation. However, upon discharging (Figure 5d), the Fe–O peak intensity and position are almost fully restored, closely matching the initial spectrum at 2.5 V. This suggests that the redox transition between Fe^2+^ and Fe^3+^ is highly reversible in the presence of nano-LLZTO. The improved reversibility is attributed to several synergistic effects introduced by LLZTO. First, nano-LLZTO facilitates fast and uniform Li^+^ migration across the cathode, as confirmed by GITT measurements, promoting complete and homogeneous lithiation during discharge. Second, efficient Li^+^ transport helps prevent localized overoxidation or under-lithiation, reducing the formation of disordered FeO_6_ environments. Third, LLZTO may act as a buffer layer at LiFePO_4_ particle boundaries, alleviating mechanical strain and preserving the local Fe–O coordination during repeated cycling. These structural advantages are reflected in the greater intensity recovery and peak position restoration of the Fe–O shell in Figure 5d, compared to the irreversible behavior seen in the pristine LiFePO_4_ (Figure 5b). Although absolute Fe–O bond distances cannot be precisely determined from the R-space spectra alone, the consistent trend in peak evolution clearly indicates that nano-LLZTO plays a crucial role in maintaining structural reversibility. In both systems, the Fe–O coordination undergoes reversible changes during cycling, but the extent of reversibility differs significantly. The pristine LiFePO_4_ suffers from partial structural irreversibility, whereas the LiFePO_4_/nano-LLZTO composite exhibits robust recovery of the local Fe environment. This finding highlights the dual function of nano-LLZTO: not only as a lithium-ion conductor that improves electrochemical performance, but also as a structural stabilizer that protects the Fe–O framework from irreversible damage. These features make nano-LLZTO a promising additive for improving the cycle life and reliability of solid-state lithium-ion batteries. These results demonstrate that the incorporation of nano-LLZTO not only accelerates ion transport but also promotes deeper and more uniform redox conversion within the LiFePO_4_ matrix—an effect critical for achieving high-performance and long-life solid-state batteries.

### 3.5 Enhanced Li^+^ Diffusion in Cathodes and across the Cathode–Electrolyte Interface

For the two solid-state batteries assembled with pristine LiFePO_4_ and composite LiFePO_4_/nano-LLZTO cathodes, the *D*_Li_^+^, particularly within the cathode and solid electrolyte regions, was determined using GITT.^43^ Figure 6a shows the GITT voltage profiles during charge and discharge at a rate of 0.1C. Based on the voltage responses recorded during and after each current pulse (Figure S16), and the observed linear relationship between *E*(*V*) and $\sqrt{t}$ across the investigated voltage range, *D*_*Li* ^+^_ was calculated using eq 3:^44^

3 $$D_{{Li}^{+}}=\frac{4}{\pi \lambda }{\left(\frac{n_{m}V_{m}}{S}\right)}^{2}{\left(\frac{\Delta E_{s}}{\Delta E_{t}}\right)}^{2}$$

where λ is the pulse duration, *n*_*m*_ is the molar amount of cathode active material, *V*_*m*_ is its molar volume, *S* is the electrode–electrolyte contact area, Δ*E*_*s*_ is the steady-state voltage change after the current pulse, and Δ*E*_*t*_ is the voltage change during the pulse (excluding IR drop). The resulting values are compared in Figure 6b. The average diffusion coefficients for the pristine LiFePO_4_ and LiFePO_4_/nano-LLZTO batteries are 3.9 × 10^–12^ and 5.8 × 10^–12^ cm^2^ s^–1^, respectively, indicating that Li^+^ transport is more favorable in the battery with the composite cathode.

**Figure 6:**
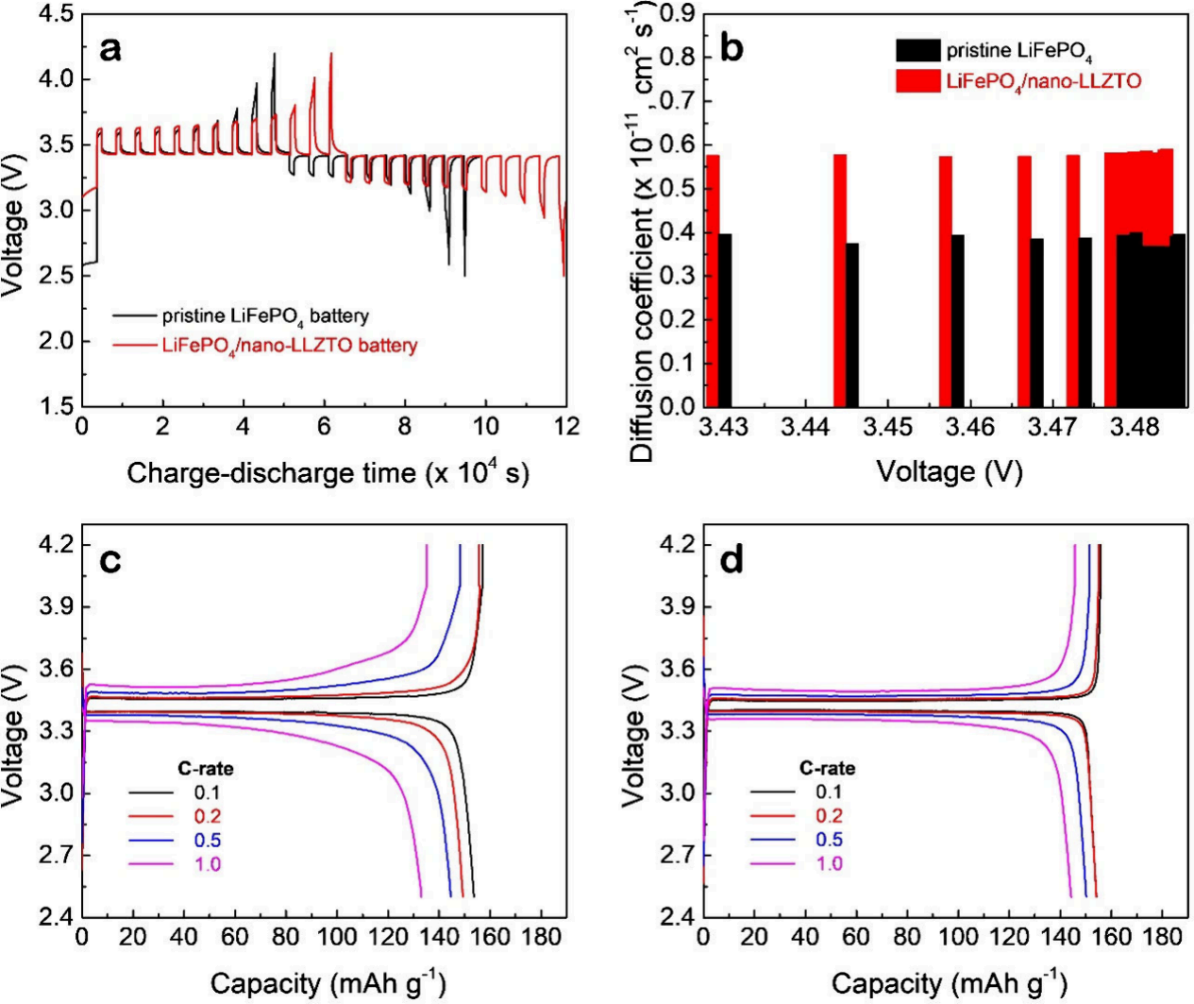
(a) GITT curves during charge–discharge and (b) the corresponding calculated Li^+^ diffusion coefficients for SSLBs with pristine LiFePO_4_ and LiFePO_4_/nano-LLZTO cathodes. Galvanostatic charge–discharge profiles at various C-rates (0.1–1C) for SSLBs with (c) pristine LiFePO_4_ and (d) LiFePO_4_/nano-LLZTO cathodes.

This enhancement can be attributed to the effective shortening of Li^+^ transport pathways enabled by the homogeneous distribution of LLZTO nanoparticles within the cathode. Instead of Li^+^ ions migrating over long distances through the polymer electrolyte or accessing isolated LiFePO_4_ surfaces, the presence of nano-LLZTO creates multiple short-range, high-conductivity pathways. The resulting reduction in effective Li^+^ transport tortuosity—that is, the geometric complexity of Li^+^ migration pathways through interconnected LLZTO domains and ceramic–polymer interfacial regions—facilitates faster and more uniform Li^+^ access to LiFePO_4_ particle surfaces.^45^

Importantly, the intimate contact between nano-LLZTO and LiFePO_4_ particles reduces interfacial impedance for Li^+^ transport within the composite cathode, facilitating more efficient ion exchange between the active material and the ion-conductive phase. Additionally, the presence of nano-LLZTO throughout the cathode enhances ionic connectivity across the macroscopic interface between the cathode layer and the composite solid electrolyte film, promoting continuous Li^+^ transport throughout the full cell architecture. This observation is consistent with the previous discussion in Figure 2b and d. As a result, the electrochemical lithiation and delithiation processes become more efficient, reducing polarization and overpotential, and enabling higher utilization of the active material. The improved ionic network also contributes to a more uniform Li^+^ flux distribution, helping to mitigate localized degradation and capacity fading.^46,47^ In essence, nano-LLZTO acts as a distributed ionic highway that compensates for the inherently low *D*_Li_^+^ of LiFePO_4_, enhances the bulk ionic conductivity of the composite, and minimizes both interfacial resistance and transport bottlenecks at LiFePO_4_ particle interfaces. These combined effects lead to faster Li^+^ exchange kinetics, greater performance stability, and potentially improved rate capability, which will be examined in subsequent experiments.

### 3.6 Rate Capability Performance

To evaluate the rate capability of the two solid-state batteries, galvanostatic charge–discharge tests were performed at C-rates of 0.1C, 0.2C, 0.5C, and 1C at 25 °C, as shown in Figure 6c and d. The pristine LiFePO_4_ battery (Figure 6c) exhibits a gradual capacity drop from 154 mAh g^–1^ at 0.1C to 150, 145, and 133 mAh g^–1^ as the C-rate increases. In contrast, the battery with the LiFePO_4_/nano-LLZTO composite cathode (Figure 6d) demonstrates superior rate performance, maintaining a more stable capacity that only slightly decreases from 154 mAh g^–1^ to 154, 150, and 144 mAh g^–1^ across the same C-rate range.

Additionally, the pristine LiFePO_4_ battery exhibits a more pronounced increase in the voltage gap between the charge and discharge profiles as the C-rate increases, indicating greater polarization. In contrast, the LiFePO_4_/nano-LLZTO battery maintains significantly lower voltage hysteresis across all rates. This trend clearly reflects the higher concentration polarization in the pristine LiFePO_4_ system, which is consistent with earlier observations in Figures 2 and 3. More importantly, it aligns well with the findings from the GITT analysis (Figure 6a and b), where the LiFePO_4_/nano-LLZTO battery exhibited a higher *D*_Li_^+^ than the pristine system. The enhanced diffusivity facilitates faster ion transport and reduces mass transport limitations at elevated current densities, thus supporting more complete lithiation/delithiation even under faster charge–discharge conditions. Together, these results confirm that incorporating nano-LLZTO into the cathode not only improves ionic pathways and interfacial transport but also directly contributes to the battery’s robust high-rate performance.

### 3.7 Effect of LLZTO Particle Size on Li^+^ Transport and Electrochemical Performance

FEM simulations suggest that reducing the LLZTO particle size from the microscale to the nanoscale substantially alters the Li^+^-transport landscape in the composite cathode. In particular, the nano-LLZTO model yields a more uniform and higher-magnitude Li^+^ flux distribution across the cathode microstructure compared with micro-LLZTO, implying a more continuous ion-transport network and mitigated transport bottlenecks inside the electrode. This trend is consistent with experimental ionic conductivity measurements of LLZTO/PVDF-HFP composite solid electrolytes (Figure S10 and Table S3), where the nano-LLZTO-containing electrolyte exhibits a higher conductivity (6.48 × 10^–5^ S cm^–1^) than its micro-LLZTO counterpart (1.03 × 10^–5^ S cm^–1^). The agreement between simulation and experimental conductivity supports the interpretation that nanosized LLZTO more effectively promotes Li^+^ migration through percolative pathways within the solid electrolyte and, by extension, within the composite cathode.

To examine how LLZTO particle size affects electrode properties, micro-LLZTO and nano-LLZTO were individually incorporated into LiFePO_4_ to fabricate LiFePO_4_/micro-LLZTO and LiFePO_4_/nano-LLZTO composite cathodes. Because LLZTO is a poor electronic conductor, introducing LLZTO could potentially dilute the electronic percolation network of LiFePO_4_. Indeed, the electronic conductivity decreases from 3.6 × 10^–3^ S cm^–1^ for pristine LiFePO_4_ composite electrode to 1.9 × 10^–3^ S cm^–1^ for LiFePO_4_/micro-LLZTO composite electrode and 1.2 × 10^–3^ S cm^–1^ for LiFePO_4_/nano-LLZTO composite electrode (Figures S8 and S9; Table S2). Notably, however, all values remain on the same order of magnitude, indicating that the addition of LLZTO does not catastrophically compromise electron transport. Therefore, the performance differences observed below are more reasonably attributed to changes in ionic transport and interfacial resistance, rather than to a dominant limitation in electronic conductivity.

The electrochemical consequences of LLZTO particle size are clearly reflected in the rate capability and cycling stability of SSLBs (Figure 7). For the LiFePO_4_/micro-LLZTO cathode, the discharge capacities at 0.1, 0.2, 0.5, and 1C are 156, 155, 145, and 131 mAh g^–1^, respectively (Figure 7a). In contrast, the LiFePO_4_/nano-LLZTO cathode delivers 154, 154, 150, and 144 mAh g^–1^ at the corresponding C-rates (Figure 7b). While both cells show comparable capacities at low rates (0.1–0.2C), the advantage of nano-LLZTO becomes pronounced as the current increases, demonstrating superior high-rate performance. In addition, the galvanostatic profiles of the nano-LLZTO cell exhibit a smaller polarization/IR drop, suggesting a lower overall cell resistance. This reduced polarization is consistent with enhanced Li^+^ transport in the nano-LLZTO-containing cathode and electrolyte, where improved ion percolation and more effective interfacial ion conduction can decrease concentration gradients and charge-transfer/transport overpotentials during fast cycling.

**Figure 7:**
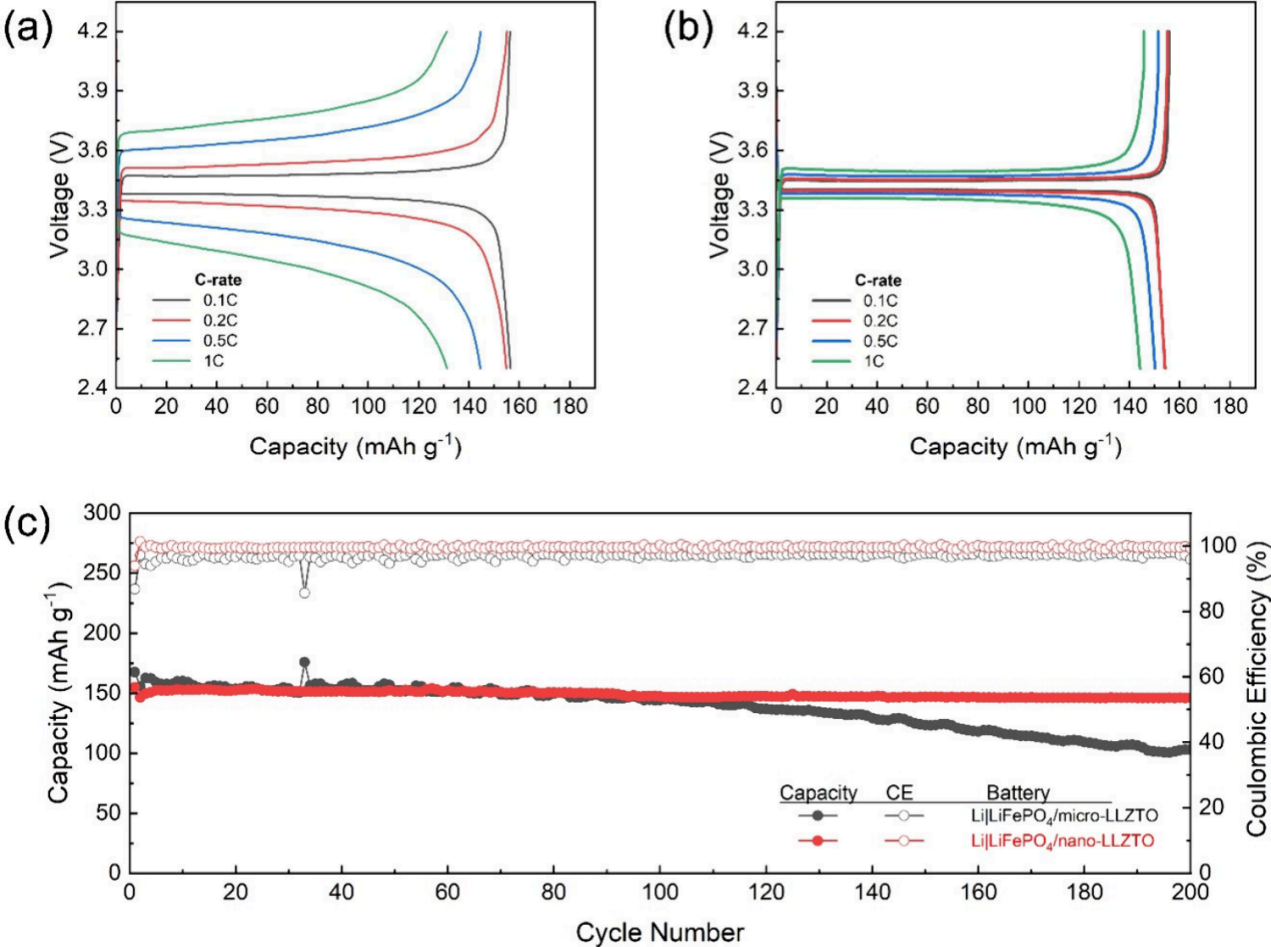
Galvanostatic charge–discharge voltage profiles of SSLBs with (a) LiFePO_4_/micro-LLZTO and (b) LiFePO_4_/nano-LLZTO composite cathodes charged and discharged at various C-rates (0.1–1C). (c) Cycle-life performance and Coulombic efficiency of SSLBs with LiFePO_4_/micro-LLZTO and LiFePO_4_/nano-LLZTO composite cathodes charged and discharged at 0.2C.

Cycle-life performance further highlights the beneficial role of nanosized LLZTO (Figure 7c). The cell with micro-LLZTO shows a gradual capacity decay during repeated cycling, although its stability remains improved relative to the pristine LiFePO_4_ cell (Figure 2a), indicating that even microscale LLZTO can partially alleviate ion-transport limitations. By comparison, the nano-LLZTO-based cell maintains markedly more stable cycling with no obvious capacity fading over 200 cycles. This improvement is also reflected in the initial reversible capacity and Coulombic efficiency (CE). The initial reversible capacity increases from 86.8% (micro-LLZTO) to 93.9% (nano-LLZTO), implying reduced irreversible processes and improved utilization of active material in the early cycles. Moreover, the average CE in subsequent cycles increases from 96.9% (micro-LLZTO) to 99.6% (nano-LLZTO), indicating more reversible Li^+^ storage and suppressed parasitic reactions, which are often exacerbated by poor interfacial contact and heterogeneous current/ion distributions.

To elucidate the structural origin of the performance enhancement, cross-sectional FIB–SEM and EDS mapping were conducted for both composite cathodes (Figure 8). For the LiFePO_4_/micro-LLZTO electrode (Figure 8a–d), La mapping reveals that micro-LLZTO exists as relatively large, localized domains rather than forming a uniform ion-conducting coating or network around LiFePO_4_. Such a morphology limits the ability of LLZTO to bridge LiFePO_4_ particles and to provide continuous Li^+^ transport pathways across the composite. Consequently, the electrode likely contains regions where LiFePO_4_ particles are poorly coupled to ion-conducting phases, leading to heterogeneous reaction distribution, higher polarization at elevated rates, and progressive capacity decay upon cycling. In sharp contrast, the LiFePO_4_/nano-LLZTO electrode (Figure 8e–h) shows a much more homogeneous La signal distributed throughout the cathode microstructure. This indicates that nanosized LLZTO can more effectively disperse and decorate the LiFePO_4_ particle surfaces, thereby increasing the interfacial contact area between the active material and the ion-conducting phase. As schematically illustrated in Figure 8i and j, nano-LLZTO is expected to form more continuous ion-conducting pathways and to reduce local Li^+^ transport distances within the composite. Such improved ionic connectivity rationalizes (i) the reduced polarization in galvanostatic profiles, (ii) the enhanced capacity retention at high C-rates, and (iii) the superior cycling stability and higher CE. Overall, these results demonstrate that downsizing LLZTO to the nanoscale is an effective strategy to engineer the cathode ion-transport network and interfacial ion conduction, thereby enabling high-rate and stable operation of LiFePO_4_-based SSLBs.

**Figure 8:**
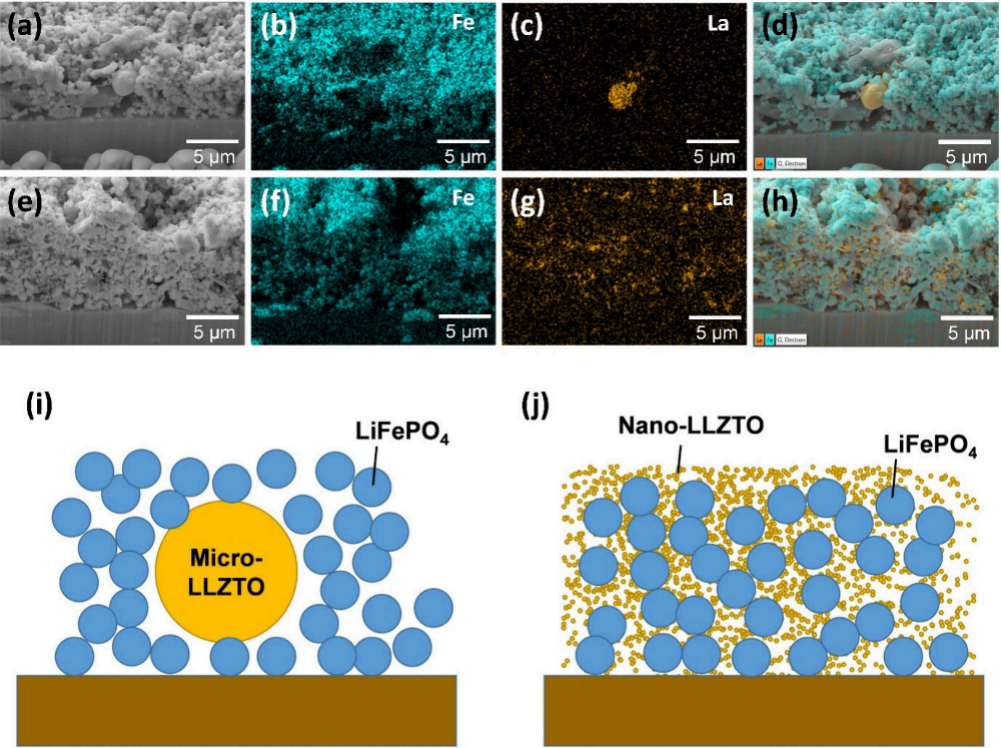
Cross-sectional FIB–SEM images and elemental mappings of LiFePO_4_/LLZTO composite electrodes. (a–d) LiFePO_4_/micro-LLZTO composite electrode: (a) cross-sectional FIB–SEM image, elemental mapping images of (b) Fe and (c) La, and (d) corresponding EDS overlay. (e–h) LiFePO_4_/nano-LLZTO composite electrode: (e) cross-sectional FIB–SEM image, elemental mapping images of (f) Fe and (g) La, and (h) corresponding EDS overlay. Schematic illustrations of (i) LiFePO_4_/micro-LLZTO and (j) LiFePO_4_/nano-LLZTO composite cathodes.

## 4 Conclusions

In this study, we demonstrated that incorporating nano-LLZTO particles into a LiFePO_4_ cathode significantly enhances the electrochemical performance of SSLBs. FEM simulations revealed that reducing the LLZTO particle size from the microscale to the nanoscale substantially improves Li^+^ flux uniformity and magnitude across the composite cathode, attributed to the formation of more continuous and efficient Li^+^-conductive pathways. Based on these insights, nano-LLZTO was selected for composite cathode fabrication. Experimentally, the SSLB with the LiFePO_4_/nano-LLZTO cathode exhibited markedly enhanced cycling stability and rate capability compared with both the pristine and micro-LLZTO counterparts. While the pristine system failed after ∼100 cycles, and the micro-LLZTO cell exhibited gradual capacity decay, the nano-LLZTO-based cell maintained stable operation for over 200 cycles with 95% capacity retention and an average Coulombic efficiency of 99.6%. Charge–discharge and CV profiles confirmed reduced polarization and voltage hysteresis, consistent with improved ionic transport and lower interfacial resistance. EIS analysis further supported these findings, revealing significantly reduced charge-transfer and diffusional resistances, indicating enhanced ionic connectivity and interfacial stability. Structural analyses corroborated these electrochemical results. In situ XANES and EXAFS demonstrated a more reversible Fe^2+^/Fe^3+^ redox transition and a more stable local Fe coordination in the nano-LLZTO composite. FIB–SEM/EDS mapping confirmed a more homogeneous LLZTO distribution and continuous Li^+^-conducting networks in the nano-LLZTO cathode, explaining its superior performance. GITT results showed higher Li^+^ diffusivity, validating the facilitation of ion transport by nanosized LLZTO. Overall, downsizing LLZTO particles effectively optimizes the cathode’s ion-transport network, interfacial architecture, and redox reversibility. This work establishes nanoscale LLZTO incorporation as a robust strategy for overcoming transport limitations and achieving high-rate, long-life operation in LiFePO_4_-based SSLBs.

## Supplementary Material









## Notes

FEM simulation video of Li^+^ flux distribution in pristine LiFePO_4_ composite electrodes during battery discharging (MP4)
FEM simulation video of Li^+^ flux distribution in LiFePO_4_/micro-LLZTO composite electrodes during battery discharging (MP4)
FEM simulation video of Li^+^ flux distribution in LiFePO_4_/nano-LLZTO composite electrodes during battery discharging (MP4)
Numerical simulation parameters configured in COMSOL; 2D models and Li^+^ flux of FEM simulations; simulated Li^+^ flux as a function of discharging time in regions of LLZTO particles distributed within the cathode; XRD patterns, SEM images, and TEM images of LLZTO; viscosity as a function of shear rate for cathode slurries; EIS of its Li|composite solid electrolyte|Li symmetric cell; electronic conductivities of LiFePO_4_/LLZTO composite electrodes; SEM and EDS results for electrolyte and electrodes; DRT plots derived from EIS spectra of SSLIBs; GITT results for SSLIBs; cross-sectional FIB–SEM images and elemental mappings of LiFePO_4_/LLZTO composite electrodes (PDF)

## References

[1] Pichaimuthu K., Nishchith B. S., Braga M. H. (2025). Sustainable, high power Li-metal
battery with LiFePO_4_ and activated carbon from terpene-rich
orange peel composite cathode. J. Energy Storage.

[2] Zhang W.-J. (2011). Structure
and performance of LiFePO_4_ cathode materials: A review. J. Power Sources.

[3] Nekahi A., Kumar M. R. A., Li X., Deng S., Zaghib K. (2024). Sustainable
LiFePO_4_ and LiMn_x_Fe_1‑x_PO_4_ (x = 0.1–1) cathode materials for lithium-ion batteries:
A systematic review from mine to chassis. Mater.
Sci. Eng. R: Rep.

[4] Li Z., Huang J., Liaw B. Y., Zhang J. (2017). On state-of-charge
determination for lithium-ion batteries. J.
Power Sources.

[5] Zheng H., Chai L., Song X., Battaglia V. (2012). Electrochemical
cycling behavior of LiFePO_4_ cathode charged with different
upper voltage limits. Electrochim. Acta.

[6] Zhao W., Wang H., Dong Q., Shao H., Zhang Y., Tang Y., Shen Y., Chen L. (2025). Mechanical stable composite
electrolyte for solid-state lithium metal batteries. Chem. Eng. J..

[7] Srivastava M., Kumar M R A., Ahmed S., Zaghib K. (2025). Exploring oxide cathodes
for Li-ion batteries: From mineral mining to active material production. J. Power Sources.

[8] Zuo W., Xiao Z., Zarrabeitia M., Xue X., Yang Y., Passerini S. (2022). Guidelines
for Air-Stable Lithium/Sodium Layered Oxide
Cathodes. ACS Mater. Lett..

[9] Xu D., Chu X., He Y.-B., Ding Z., Li B., Han W., Du H., Kang F. (2015). Enhanced performance of interconnected LiFePO_4_/C microspheres
with excellent multiple conductive network and subtle
mesoporous structure. Electrochim. Acta.

[10] Prosini P. P., Carewska M., Scaccia S., Wisniewski P., Pasquali M. (2003). Long-term cyclability of nanostructured
LiFePO_4_. Electrochim. Acta.

[11] Shanbedi M., Shahali H., Polycarpou A. A., Amiri A. (2025). Advances and future
prospects of low-temperature electrolytes for lithium-ion batteries. EES Batteries.

[12] Li J., Hu H., Zhu J., Ma X., Hu Y., Zhang H., Liu F., Zhang S., Ji X. (2025). Solid Polymer Electrolyte with Compatible
Cathode-Electrolyte Interfacial Design Enabling Lithium Metal Batteries
Operation at 4.8 V with Long Cycle Life. Adv.
Mater..

[13] Zhao Z., Wen Z., Liu X., Yang H., Chen S., Li C., Lv H., Wu F., Wu B., Mu D. (2021). Tuning a compatible
interface with LLZTO integrated on cathode material for improving
NCM811/LLZTO solid-state battery. Chem. Eng.
J..

[14] Hong S.-B., Lee Y.-J., Kim U.-H., Bak C., Lee Y. M., Cho W., Hah H. J., Sun Y.-K., Kim D.-W. (2022). All-Solid-State
Lithium Batteries: Li^+^-Conducting Ionomer Binder for Dry-Processed
Composite Cathodes. ACS Energy Lett..

[15] Kumchompoo J., Lee J.-T., Li C.-C. (2024). How dispersed LLZTO enhances ionic
conductivity in LiFePO_4_ composite cathodes for solid-state
batteries. J. Energy Storage.

[16] Li C.-C. (2025). Dispersants
and particle dispersion uniformity in lithium batteries: from slurry
formulation to solid-state design. Energy Storage
Mater..

[17] Pan C.-Y., Kuo G.-L., Li C.-C. (2025). Solid Electrolytes
and Dendrite Dynamics
in Solid-State Lithium–Sulfur Batteries. ACS Appl. Mater. Interfaces.

[18] Shi Y., Cai Y., Zhao J., Wu T., Xue X., Lin T., Lin P., Wang C., Peng H. (2023). 3D lithiophilic framework fixed on
the surface of LLZTO solid electrolyte shaping the contact between
Li metal and ceramic. Chem. Eng. J..

[19] Kuo G.-L., Li C.-C. (2025). Suppressing ceramic-polymer
gelation for high-solid-content of LLZTO
in composite electrolytes. J. Energy Storage.

[20] Yeh S.-M., Li C.-C. (2023). Enhancing Li^+^ transport efficiency in solid-state Li-ion
batteries with a ceramic-array-based composite electrolyte. J. Mater. Chem. A.

[21] Duan T., Cheng H., Sun Q., Liu Y., Nie W., Chu Y., Xu Q., Lu X. (2024). Reinforcing
interfacial compatibility
of LLZTO/PVDF-HFP composite electrolytes by chemical interaction for
solid-state lithium metal batteries. J. Power
Sources.

[22] Wang Y., Chen Z., Wu Y., Li Y., Yue Z., Chen M. (2023). PVDF-HFP/PAN/PDA@LLZTO Composite
Solid Electrolyte Enabling Reinforced
Safety and Outstanding Low-Temperature Performance for Quasi-Solid-State
Lithium Metal Batteries. ACS Appl. Mater. Interfaces.

[23] Lee K.-W., Yeh S.-M., Ni K.-H., Li C.-C. (2023). Enhancing electrochemical
performance of solid-state Li-ion batteries with composite electrolytes
of fibrous LLZTO and PVDF-HFP: The role of LLZTO fiber diameter. J. Energy Storage.

[24] Chen Q., Ouyang C., Liang Y., Liu H., Duan H. (2024). Composite
polymer electrolyte with vertically aligned garnet scaffolds for quasi
solid-state lithium batteries. Energy Storage
Mater..

[25] Zhang H., Klimpel M., Wieczerzak K., Dubey R., Okur F., Michler J., Jeurgens L. P. H., Chernyshov D., van Beek W., Kravchyk K. V., Kovalenko M. V. (2024). Unveiling
Surface Chemistry of Ultrafast-Sintered LLZO Solid-State Electrolytes
for High-Performance Li-Garnet Solid-State Batteries. Chem. Mater..

[26] Cheng E. J., Duan H., Wang M. J., Kazyak E., Munakata H., Garcia-Mendez R., Gao B., Huo H., Zhang T., Chen F., Inada R., Miyazaki K., Ohno S., Kato H., Orimo S.-i., Thangadurai V., Abe T., Kanamura K. (2025). Li-stuffed garnet solid
electrolytes: Current status,
challenges, and perspectives for practical Li-metal batteries. Energy Storage Mater..

[27] Yan S., Yim C.-H., Zhou J., Wang J., Abouali S., Baranova E. A., Weck A., Thangadurai V., Merati A., Abu-Lebdeh Y. (2023). Elucidating
the Origins of Rapid
Capacity Fade in Hybrid Garnet-Based Solid-State Lithium Metal Batteries. J. Phys. Chem. C.

[28] Zhang X., Xiang Q., Tang S., Wang A., Liu X., Luo J. (2020). Long Cycling Life Solid-State
Li Metal Batteries with Stress Self-Adapted
Li/Garnet Interface. Nano Lett..

[29] Lin X., Chu C., Li Z., Zhang T., Chen J., Liu R., Li P., Li Y., Zhao J., Huang Z., Feng X., Xitoe Y., Ma Y. (2021). A high-performance, solution-processable
polymer/ceramic/ionic liquid electrolyte for room temperature solid-state
Li metal batteries. Nano Energy.

[30] Goujon N., Aldalur I., Santiago A., Armand M., Martinez-Ibañez M., Zhang H. (2024). Opportunity
for lithium-ion conducting polymer electrolytes beyond
polyethers. Electrochim. Acta.

[31] D, M. ; M, U. R. , Optimization of polymer electrolytes for Li-ion batteries: focus on enhancement strategies and film casting techniques. Ionics 2025, 31 8789 10.1007/s11581-025-06509-5.

[32] van
der Stam W., Gudjonsdottir S., Evers W. H., Houtepen A. J. (2017). Switching
between Plasmonic and Fluorescent Copper Sulfide Nanocrystals. J. Am. Chem. Soc..

[33] Cho C.-S., Wu Y.-W., Liao M.-W., Chen J.-K., Li C.-C. (2024). In-situ
analysis of cathode and anode impedances to probe the performance
degradation of lithium–sulfur batteries. J. Power Sources.

[34] Wu Y.-W., Li C.-C. (2025). Electrochemical
assessment of a Li-ion full cell with cathode-anode
impedance separation via in-situ EIS-DRT and three-electrode configuration. J. Power Sources.

[35] Kumchompoo J., Li C.-C., Bolloju S., Chang Y.-L., Liao B.-C., Wiboon M., Chen P.-J., Lee J.-T. (2025). Super-theoretical
capacity of CoO/Co_3_O_4_ hybrid oxide anodes in
lithium-ion batteries via electrolyte additives. Colloids Surf., A.

[36] Wang, H. ; Hoang, B. ; Wang, F. ; Liou, S.-C. ; Wang, C. ; Rubloff, G. ; Lin, C.-F. , Oversaturated Li-FeOF solid solutions developed using LiPON interfacial coating. Mater. Adv. 2025, 6, 5892 10.1039/D5MA00338E.

[37] Lu Y., Li J., Zhao Y., Zhu X. (2019). Lithium Clustering during the Lithiation/Delithiation
Process in LiFePO_4_ Olivine-Structured Materials. ACS Omega.

[38] Larouche F., Voisard F., Amouzegar K., Houlachi G., Bouchard P., Vijh A., Demopoulos G. P. (2023). Kinetics,
Mechanism, and Optimization
Modeling of a Green LFP Delithiation Process Developed for Direct
Recycling of Lithium-Ion Batteries. Ind. Eng.
Chem. Res..

[39] Haas O., Deb A., Cairns E. J., Wokaun A. (2005). Synchrotron
X-Ray Absorption Study
of LiFePO_4_ Electrodes. J. Electrochem.
Soc..

[40] Yao W., Armstrong A. R., Zhou X., Sougrati M.-T., Kidkhunthod P., Tunmee S., Sun C., Sattayaporn S., Lightfoot P., Ji B., Jiang C., Wu N., Tang Y., Cheng H.-M. (2019). An oxalate cathode for lithium ion
batteries with combined cationic and polyanionic redox. Nat. Commun..

[41] Etesami M., Mano P., Namuangruk S., Khezri R., Gopalakrishnan M., Limphirat W., Yonezawa T., Motlagh S. R., Somwangthanaroj A., Kheawhom S. (2025). Solvent-free synthesis of FeCo alloy nanoparticle-embedded
nitrogen-doped carbon nanotubes for oxygen reduction in zinc-air batteries. Int. J. Hydrog. Energy.

[42] Ji B., Yao W., Zheng Y., Kidkhunthod P., Zhou X., Tunmee S., Sattayaporn S., Cheng H.-M., He H., Tang Y. (2020). A fluoroxalate
cathode material for potassium-ion batteries with ultra-long cyclability. Nat. Commun..

[43] Cho C.-S., Chen J.-K., Li C.-C. (2022). Construction of
an Additional Hierarchical
Porous Framework in Carbon Fabric for Applications in Energy Storage. Chem. Mater..

[44] Zou Y., Xiao Y., Tang Y., Cheng Y., Sun S.-G., Wang M.-S., Yang Y., Zheng J. (2023). Synergetic LaPO_4_ and Al_2_O_3_ hybrid
coating strengthens
the interfacial stability of LiCoO_2_ at 4.6 V. J. Power Sources.

[45] Dixit M. B., Regala M., Shen F., Xiao X., Hatzell K. B. (2019). Tortuosity
Effects in Garnet-Type Li_7_La_3_Zr_2_O_12_ Solid Electrolytes. ACS Appl. Mater.
Interfaces.

[46] Gharehkhani A., Ghorbani-vaghei R., Alavinia S. (2021). Synthesis of calixresorcarenes using
magnetic polytriazine-benzene sulfonamide-SO_3_H. RSC Adv..

[47] Lee J., Park H., Hwang J., Noh J., Yu C. (2023). Delocalized
Lithium Ion Flux by Solid-State Electrolyte Composites Coupled with
3D Porous Nanostructures for Highly Stable Lithium Metal Batteries. ACS Nano.

