# Supplementary material for: LiFePO4/Nano-LLZTO Composite Cathodes for Enhanced Performance of Solid-State Lithium Batteries
Source: ACS Appl Mater Interfaces. 2026 Feb 27;18(9):13848–60. doi: 10.1021/acsami.5c25967 (PMC12983195; doi:10.1021/acsami.5c25967)
Supplement: Supplementary file 4 [file am5c25967_si_004.pdf]

Supporting Information

$LiFePO_4$/nano-LLZTO Composite Cathodes for Enhanced Performance of Solid-State Lithium Batteries

Jaturon Kumchompoo,[a,b] Bo-Huei Yang,[b] Jintara Padchasri,[c] Pinit Kidkhunthod,[c] Jyh-Tsung Lee,[b,d,] Chia-Chen Li[a,]**

[a]Department of Materials Science and Engineering, National Tsing Hua University, Hsinchu 30013, Taiwan

[b]Department of Chemistry, National Sun Yat-Sen University, Kaohsiung 80424, Taiwan

[c]Synchrotron Light Research Institute (Public Organization), 111 University Avenue, Muang District, Nakhon Ratchasima, 30000, Thailand

[d]Department of Medicinal and Applied Chemistry, Kaohsiung Medical University, Kaohsiung 80708, Taiwan

*Corresponding authors. Tel.: 886-7-5252000. E-mail: jtlee@faculty.nsysu.edu.tw; Tel.: 886-3-5715131. E-mail: cc.li@mx.nthu.edu.tw

Keywords: Solid-state lithium battery; Composite cathode; Conductivity; LLZTO nanoparticles; Numerical simulation

Table S1. Numerical simulation parameters configured in COMSOL. [S1−S3]

Parameter	Units	Value	Description
$D_{Li^+}^{LLZTO}$	$m^2\ s^{-1}$	7.5×10^{-12}	Li^+ diffusivity in LLZTO
$D_{Li^+}^{LE}$	$m^2\ s^{-1}$	10^{-6}	Li^+ diffusivity in liquid electrolyte
$D_{Li^+}^{LiFePO4}$	$m^2\ s^{-1}$	1.8×10^{-14}	Li^+ diffusivity in $LiFePO_4$ cathode
$D_{Li^+}^{Li}$	$m^2\ s^{-1}$	$> 10^{-9}$	Li^+ diffusivity in Li metal
σ_{LLZTO}	$S\ m^{-1}$	1.8×10^{-4}	ionic conductivity of LLZTO
$\sigma_{LiFePO4}$	$S\ m^{-1}$	10^{-10}	ionic conductivity of $LiFePO_4$
$M_{Ceramic}$	wt%	10	mass ratio of LLZTO in a cathode
$M_{LiFePO4}$	wt%	75	mass ratio of $LiFePO_4$ in a cathode
$W_{Cathode}$	μm	45	width of a cathode
$\delta_{Cathode}$	μm	85	thickness of a cathode
T	^oC	298	temperature
t	s	18000	time for discharge at 0.2C

Note: The particle sizes of micro and nano-LLZTO were set to 5 μm and 200 nm, respectively.

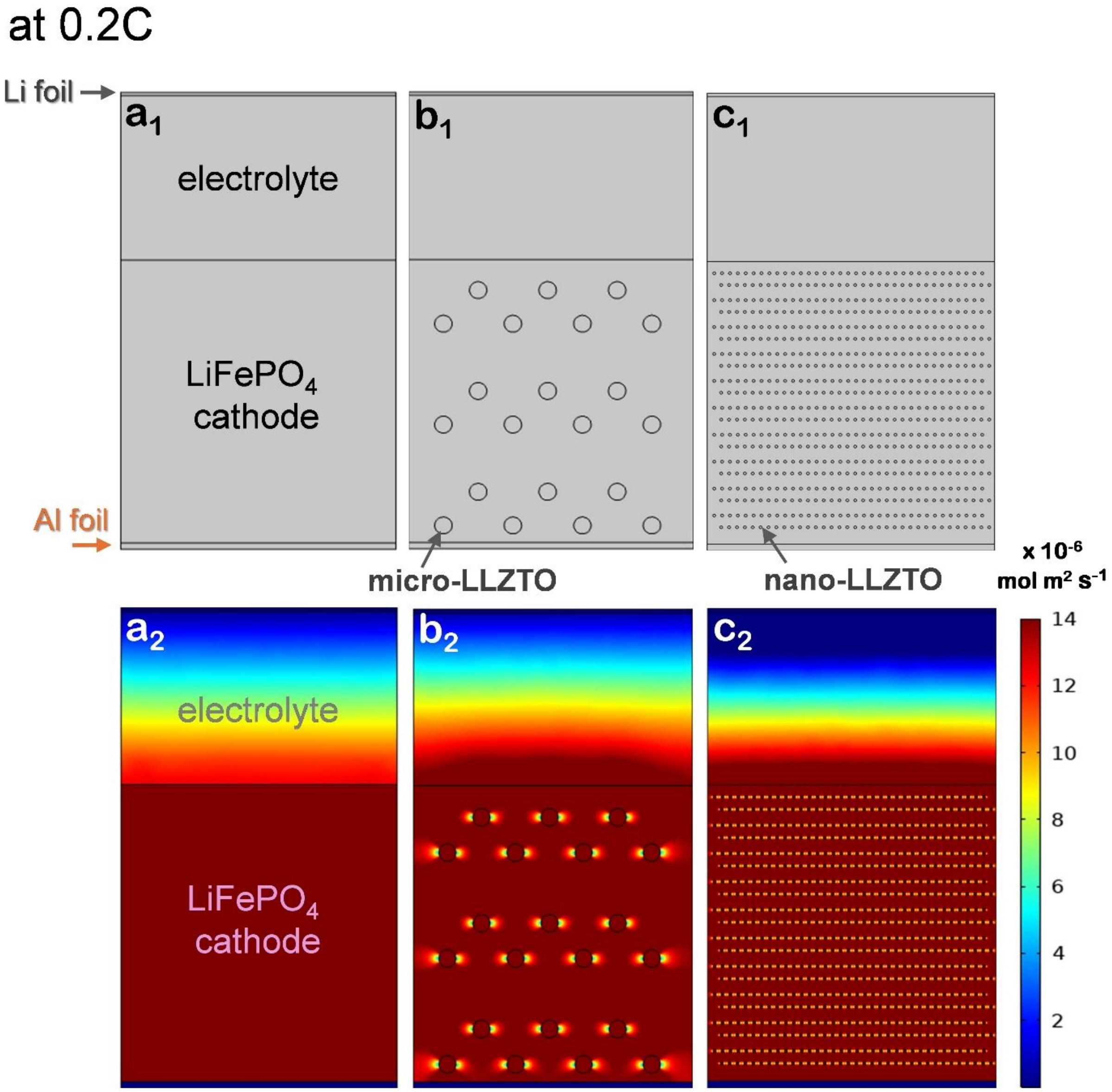

Figure S1. (a_1-c_1) 2D models and (a_2-c_2) Li^+ flux obtained during the beginning of discharge for solid-state batteries made with (a_1,a_2) pristine $LiFePO_4$ cathode and the composite cathodes of (b_1,b_2) $LiFePO_4$/micro-LLZTO and (c_1,c_2) $LiFePO_4$/nano-LLZTO particles.

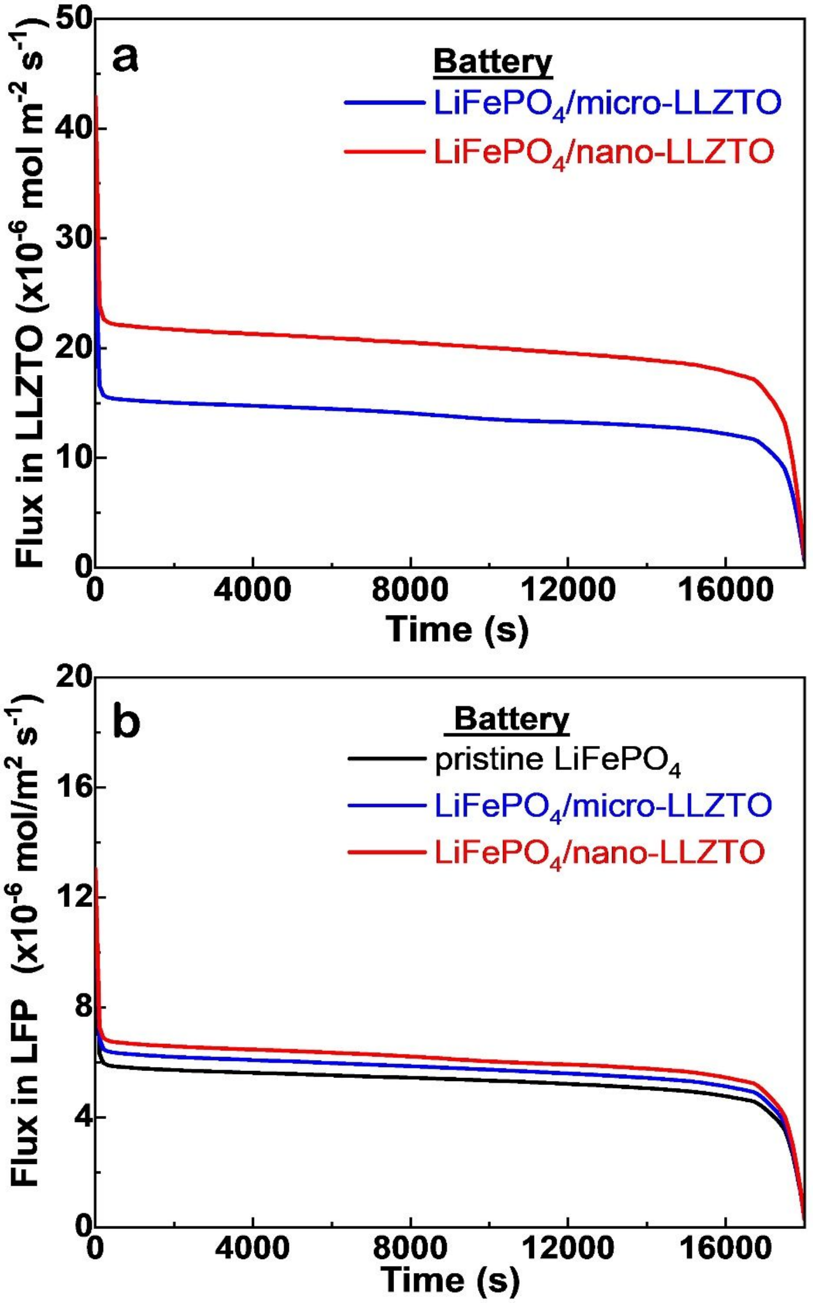

Figure S2. Simulated Li^+ flux as a function of discharging time in regions of (a) LLZTO particles distributed within the cathode and (b) the $LiFePO_4$ matrix. Note: discharging rate is 0.2C.

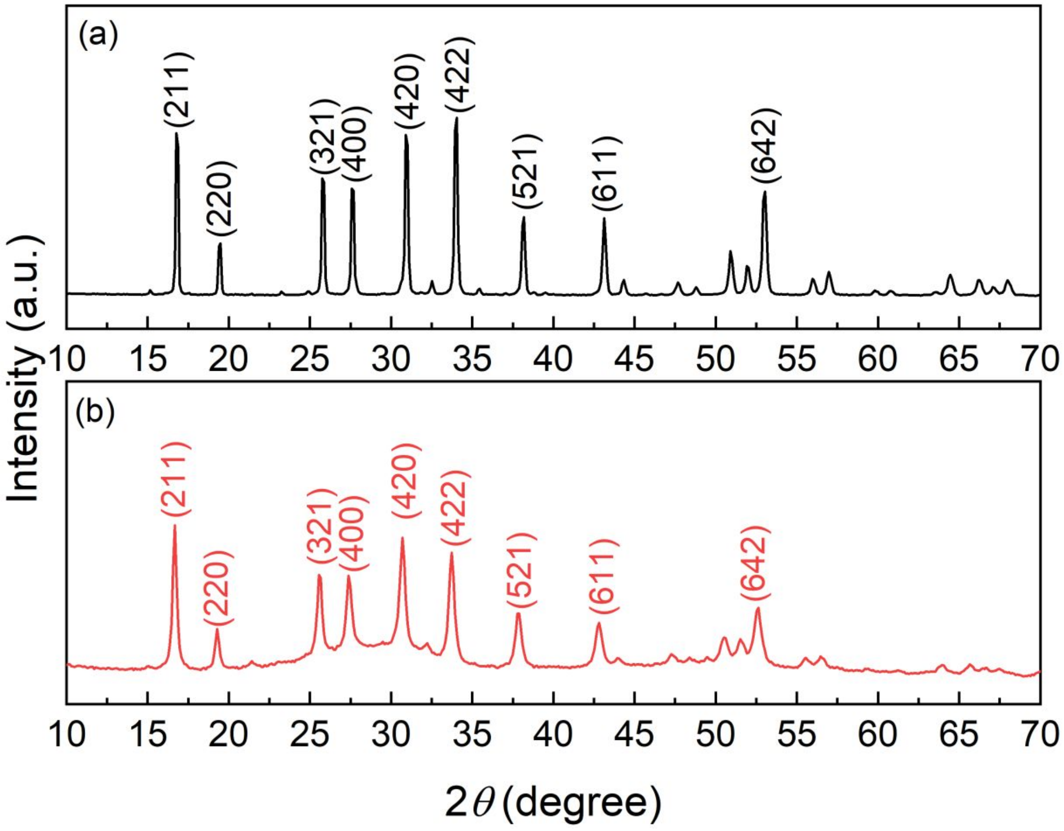

Figure S3. XRD patterns of (a) micro-sized and (b) nano-sized LLZTO particles.

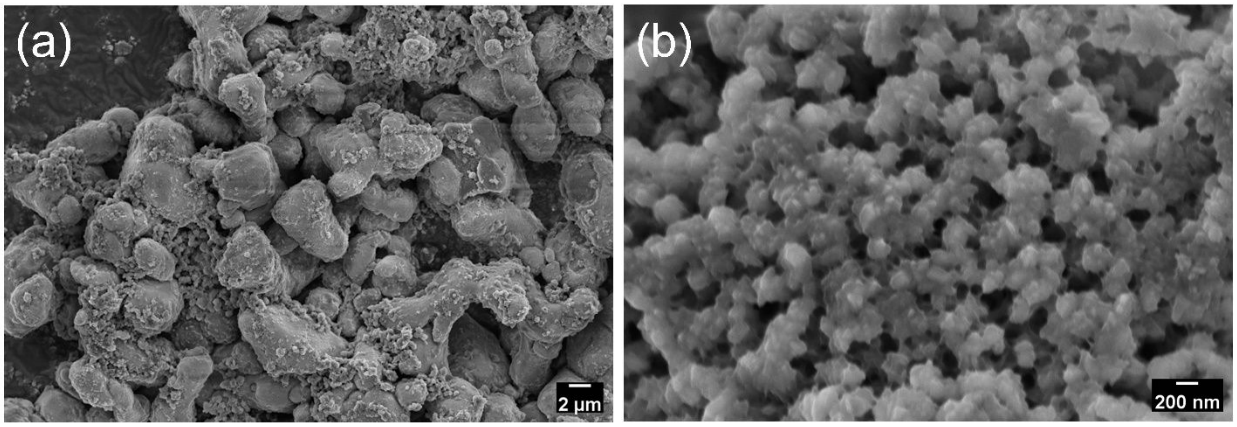

Figure S4. SEM images of (a) micro-sized and (b) nano-sized LLZTO particles.

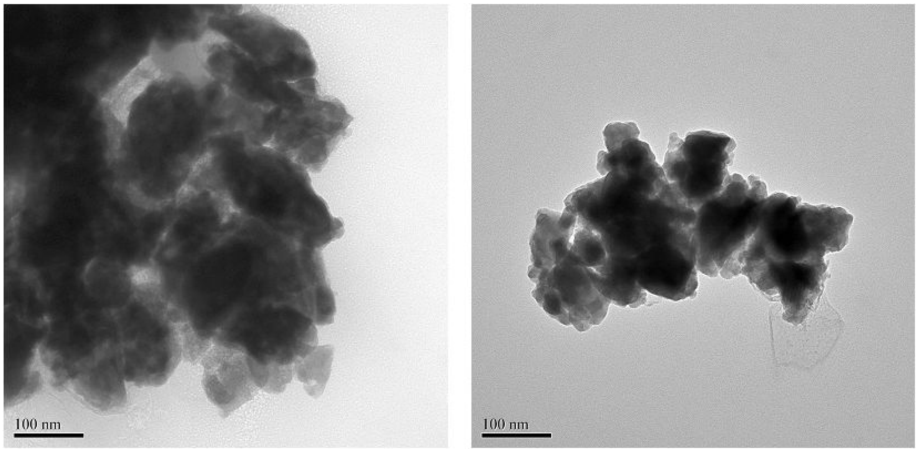

Figure S5. TEM images of the nano-LLZTO powder acquired at the same magnification from different regions.

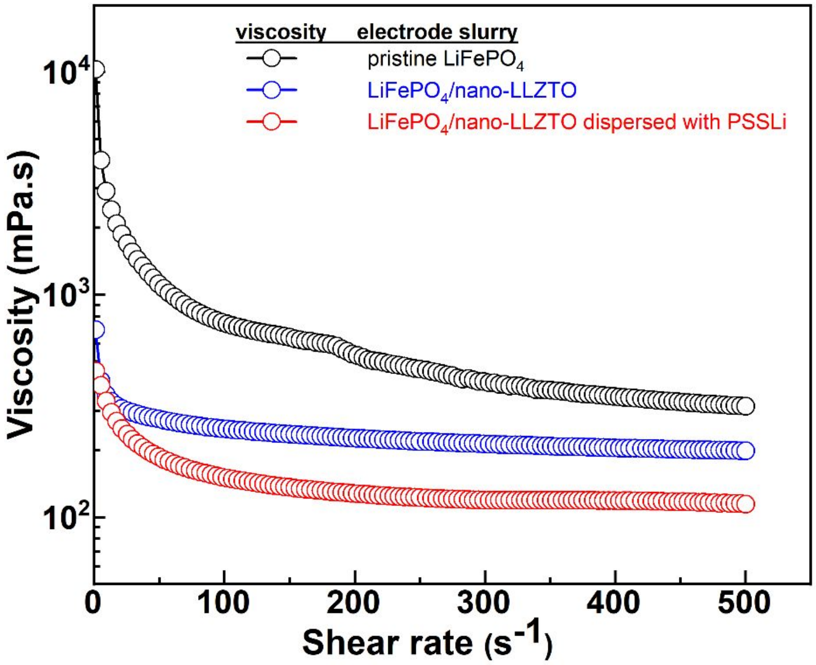

Figure S6. Viscosity as a function of shear rate for various cathode slurries of pristine $LiFePO_4$, $LiFePO_4$/nano-LLZTO, and $LiFePO_4$/nano-LLZTO dispersed with PSSLi addition.

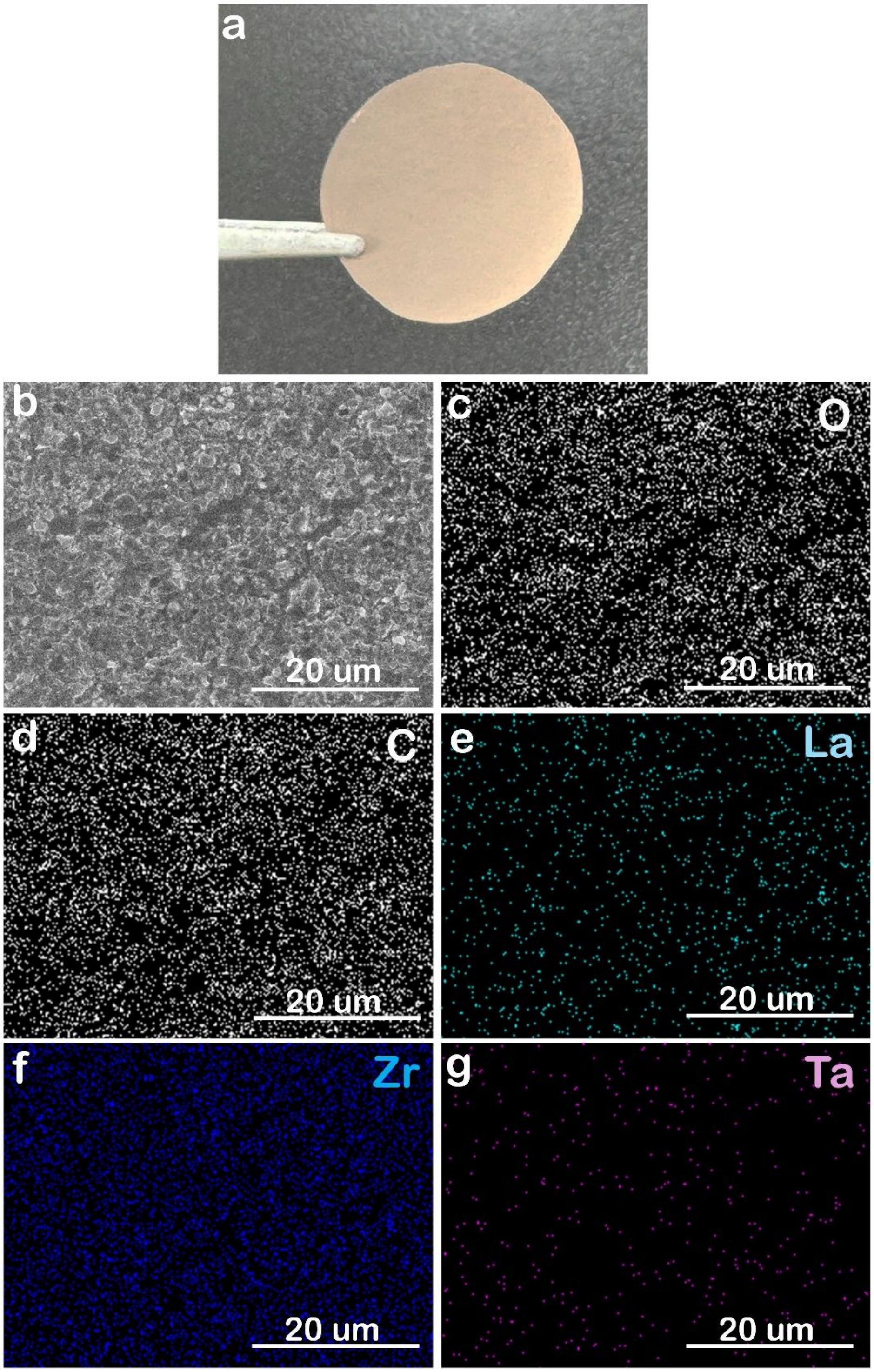

Figure S7. Fabricated composite solid electrolyte: (a) photograph, (b) SEM image, and (c-g) SEM EDS results of elements including (c) O, (d) C, (e) La, (f) Zr, and (g) Ta.

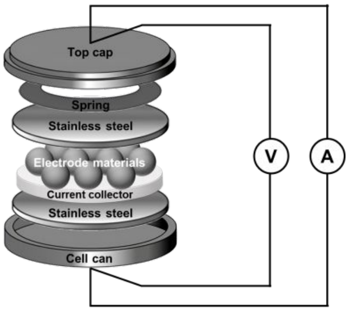

Figure S8. Schematic illustration of the electronic conductivity measurement setup for electrode materials using a coin-cell accessory. The electrode sample was sandwiched between two stainless-steel spacers and assembled into a coin cell for electrical characterization. The resistance of the samples was measured using an LCR meter (TH2829A, Changzhou Tonghui Electronic Co., Ltd, China).

Table S2. Electronic resistance, resistivity, and conductivity of pristine $LiFePO_4$ and $LiFePO_4$/LLZTO composite electrodes measured using a coin-cell configuration, where *A* is the electrode area and *L* is the electrode thickness.

	Resistance (Ohm)	Resistivity (Ohm cm)	A (cm^2)	L (cm)	Conductivity (S/cm)
**pristine $LiFePO_4$**	0.5280±0.0668	280.3364±35.4557	1.3273	0.0025	0.0036±0.0004
**$LiFePO_4$/micro-LLZTO**	1.2429±0.0834	515.5150±34.6052	1.3273	0.0032	0.0019±0.0001
**$LiFePO_4$/micro-LLZTO**	2.2616±0.1775	833.8504±65.4695	1.3273	0.0036	0.0012±0.0001

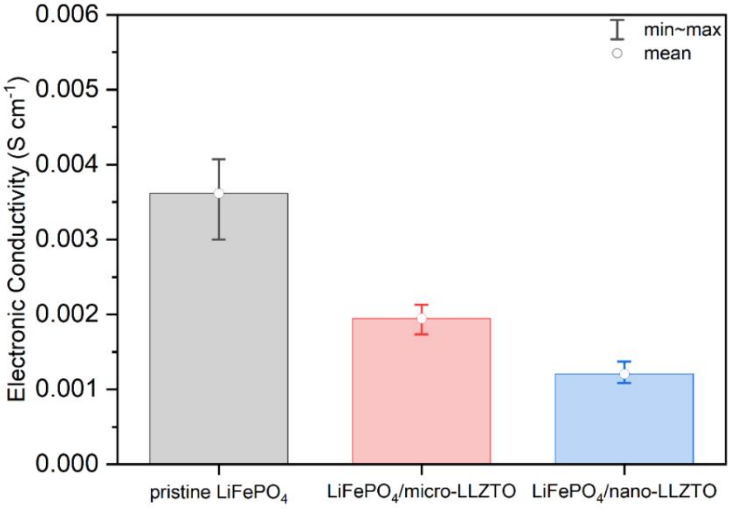

Figure S9. Electronic conductivity of pristine $LiFePO_4$, $LiFePO_4$/LLZTO, and $LiFePO_4$/LLZTO composite electrodes measured using a coin-cell configuration.

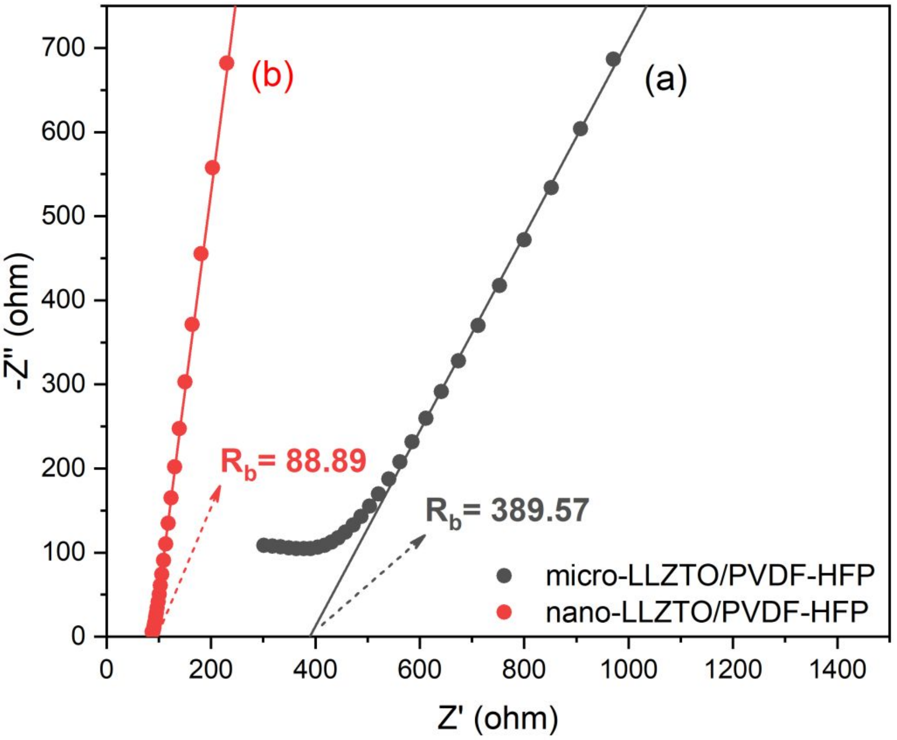

Figure S10. EIS spectra of (a) SS|micro-LLZTO/PVDF-HFP|SS and (b) SS|nano-LLZTO/PVDF-HFP|SS at 30 ^{o}C.

Table S3. Ionic conductivity of micro-LLZTO/PVDF-HFP and nano-LLZTO/PVDF-HFP determined from bulk resistance (R_b) measurements obtained from EIS spectra.

	L (cm)	R_b (ohm)	A (cm^2)	σ ($S\ cm^{-1}$)
micro-LLZTO	0.0079	443.98	1.961	1.03×10^{-5}
nano-LLZTO	0.0113	89.53	1.961	6.48×10^{-5}

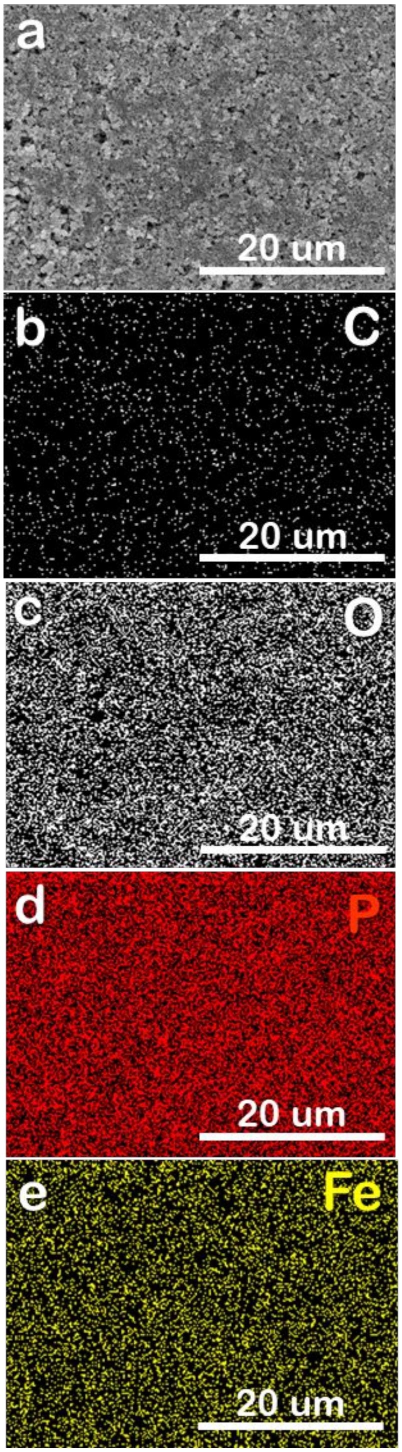

Figure S11. The fresh cathodes of (a-e) pristine $LiFePO_4$: (a) top surface SEM image, and corresponding SEM EDS results of elements including (b) C, (c) O, (d) P, and (e) Fe.

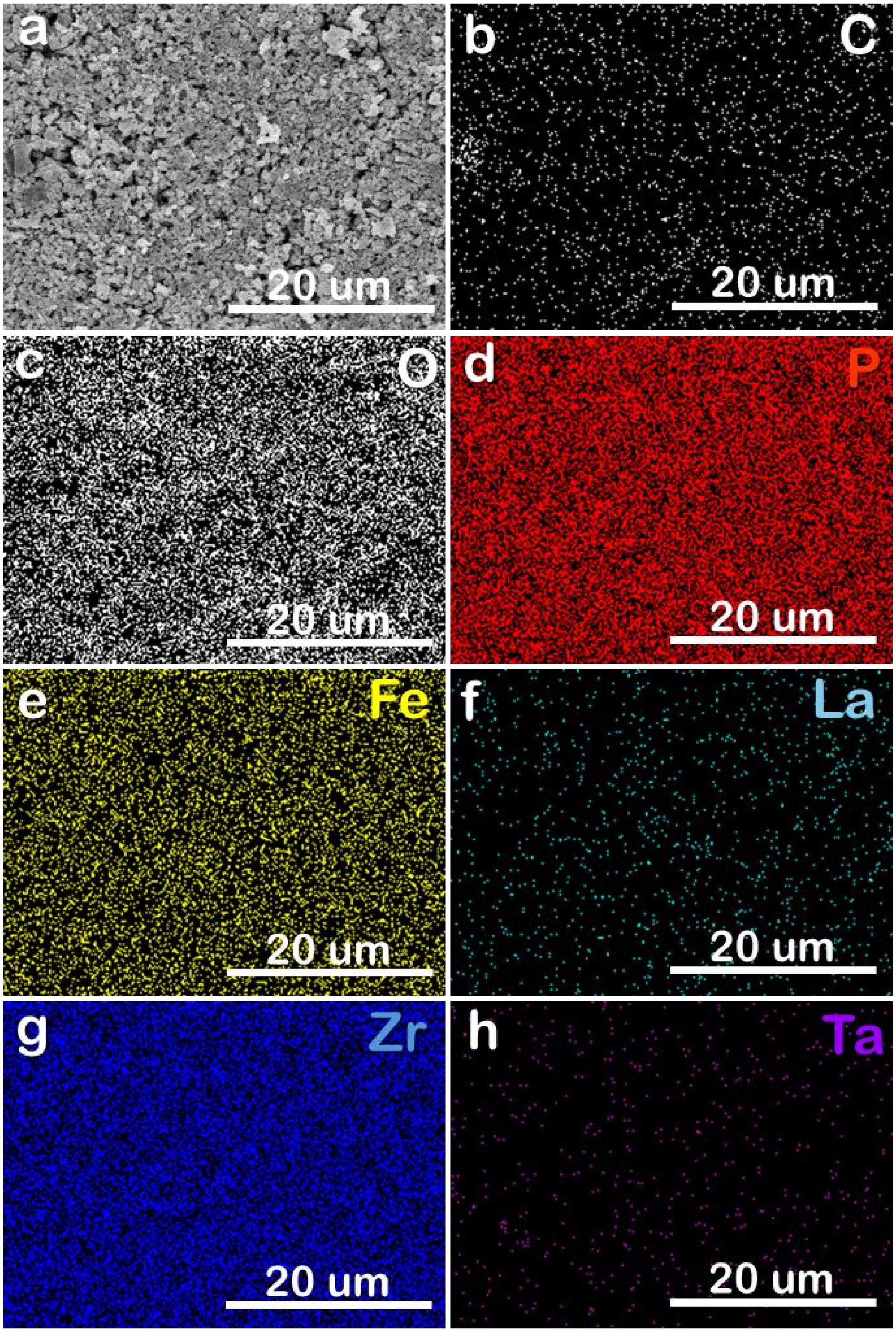

Figure S12. The fresh cathodes of (a-h) $LiFePO_4$/nano-LLZTO: (a) top surface SEM image, and corresponding SEM EDS results of elements including (b) C, (c) O, (d) P, (e) Fe, (f) La, (g) Zr, and (h) Ta.

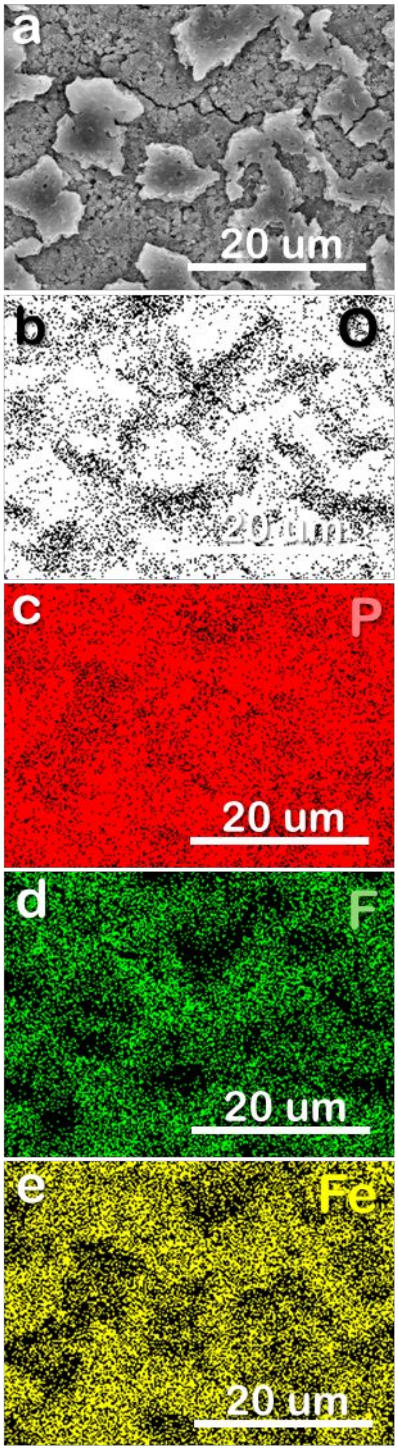

Figure S13. The cathodes received from 200 charge-discharge cycles at 0.2C of solid-state lithium-ion batteries made with pristine $LiFePO_4$ cathode: (a) top surface SEM image, and corresponding SEM EDS results of elements including (b) O, (c) P, (d) F, and (e) Fe.

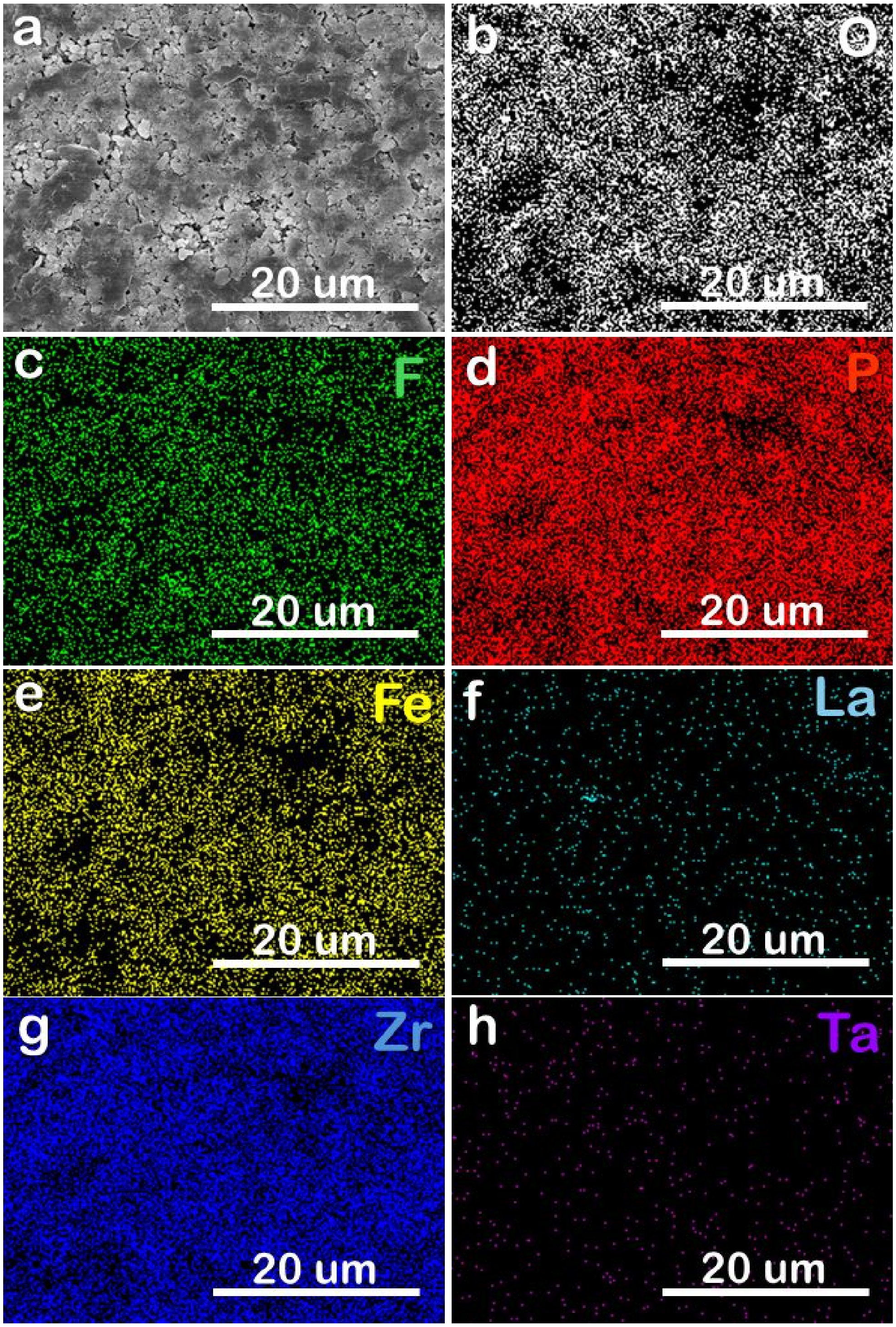

Figure S14. The cathodes received from 200 charge-discharge cycles at 0.2C of solid-state lithium-ion batteries made with $LiFePO_4$/nano-LLZTO cathode: (a) top surface SEM image, and corresponding SEM EDS results of elements including (b) C, (c) O, (d) P, (e) Fe, (f) La, (g) Zr, and (h) Ta.

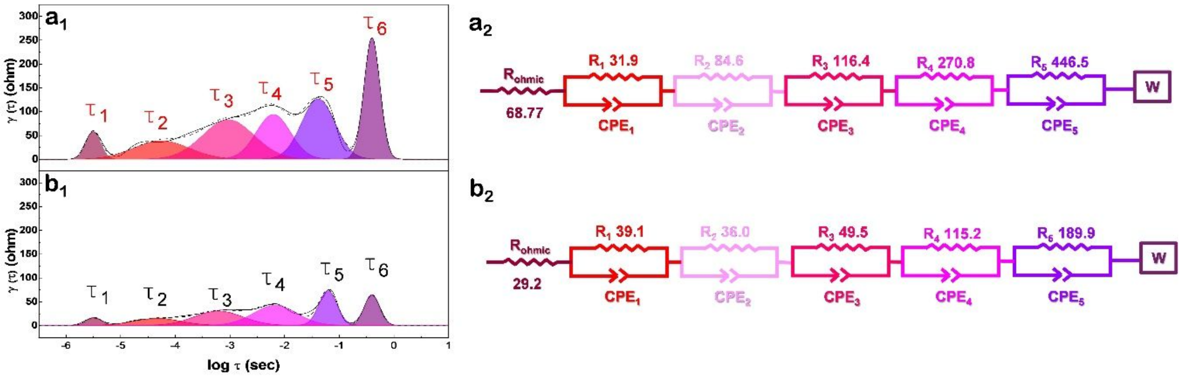

Figure S15. DRT plots derived from EIS spectra of solid-state lithium-ion batteries assembled with cathodes of (a_1) pristine $LiFePO_4$ and (b_1) $LiFePO_4$/nano-LLZTO (dispersed with PSSLi) before cycling, along with their corresponding equivalent circuit models and impedance values (a_2) pristine $LiFePO_4$ and (b_2) $LiFePO_4$/nano-LLZTO (dispersed with PSSLi).

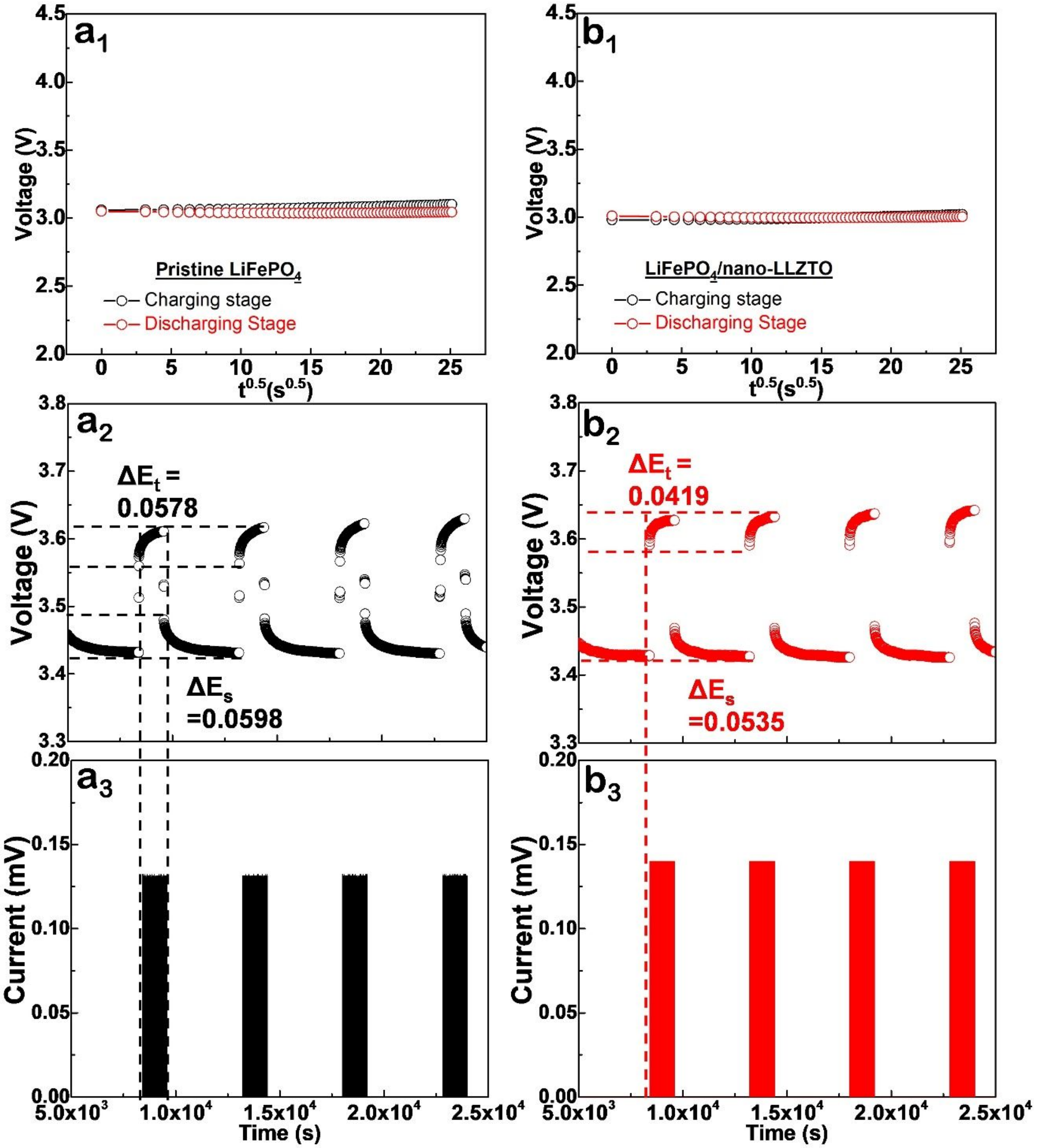

Figure S16. (a_1,b_1) Relationship between *E(V)* and $\sqrt{t}$ in GITT, (a_2-a_3,b_2-b_3) voltage and current curves, indicating ΔE_t and ΔE_s values, for solid-state lithium-ion batteries assembled with (a_1-a_3) the pristine $LiFePO_4$ cathode and (b_1-b_3) $LiFePO_4$/nano-LLZTO composite cathode.

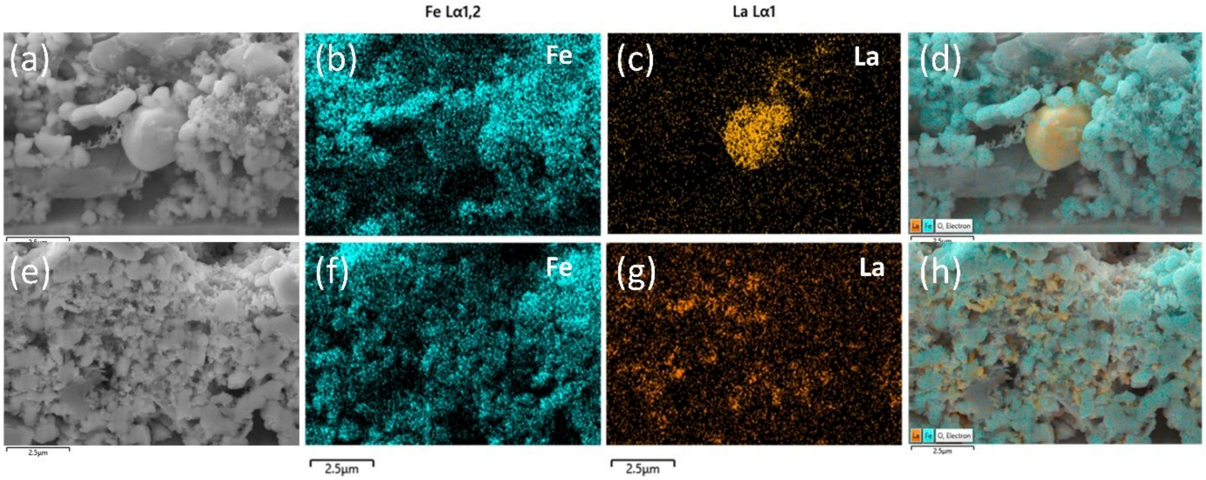

Figure S17. Cross-sectional FIB–SEM images and elemental mappings of $LiFePO_4$/LLZTO composite electrodes. (a–d) $LiFePO_4$/micro-LLZTO composite electrode: (a) cross-sectional FIB–SEM image, elemental mapping images of (b) Fe and (c) La, and (d) corresponding EDS overlay. (e–h) $LiFePO_4$/nano-LLZTO composite electrode: (e) cross-sectional FIB–SEM image, elemental mapping images of (f) Fe and (g) La, and (h) corresponding EDS overlay.

Reference:

S1. Martin, W.; Tian, Y.; Xiao, J. Understanding Diffusion Electrochemical Reduction of Li^+ Ions in Liquid Lithium Metal Batteries. *J. Electrochem. Soc.* **2021**, *168*, 060513.

S2. Nishida, T.; Nishikawa, K.; Fukunaka, Y. Diffusivity Measurement of $LiPF_6$, LiTFSI, $LiBF_4$ in PC. *ECS Trans.* **2008**, *6*, 1.

S3. Kumchompoo, J.; Lee, J.-T.; Li, C.-C. How Dispersed LLZTO Enhances Ionic Conductivity in $LiFePO_4$ Composite Cathodes for Solid-State Batteries. *J. Energy Storage* **2024**, *102*, 114215.
